# Membrane transporter progressive ankylosis protein homologue (*ANKH*/*Ank*) partially mediates senescence-derived extracellular citrate and is regulated by DNA damage, inflammation, and ageing

**DOI:** 10.3389/fragi.2025.1583288

**Published:** 2025-06-04

**Authors:** Emma Naomi James, Muy-Teck Teh, Yufeng Li, Christine Wagner-Bock, Zahra Falah Al-Khateeb, Lee Peng Karen-Ng, Terry Roberts, Linnea Synchyshyn, Amy Lewis, Ana O’Loghlen, Andrew Silver, Adina Teodora Michael-Titus, Mark Bennett, Jacob Guy Bundy, Maria Elzbieta Mycielska, Eric Kenneth Parkinson

**Affiliations:** ^1^ Centre for Oral Immunology and Regenerative Medicine, Institute of Dentistry, Barts and the London School of Medicine and Dentistry, Queen Mary University of London, London, United Kingdom; ^2^ Department of Metabolism, Digestion and Reproduction, Imperial College London, London, United Kingdom; ^3^ Universitätsklinikum Regensburg, Department of Dermatology, Regensburg, Germany; ^4^ Centre for Neuroscience, Surgery and Trauma, Blizard Institute, London, United Kingdom; ^5^ Division of Biosciences, Department of Life Sciences, Centre for Genome Engineering and Maintenance, College of Health and Life Sciences, Brunel University London, Uxbridge, United Kingdom; ^6^ Centre for Genomics and Child Health, Colorectal Cancer Genetics Group, London, United Kingdom; ^7^ Centre for Genomics and Child Health, London, United Kingdom; ^8^ Department of Life Sciences, Imperial College London, London, United Kingdom; ^9^ Department of Structural Biology, Institute of Biophysics and Physical Biochemistry, University of Regensburg, Regensburg, Germany

**Keywords:** ANKH/SLC62A1, citrate, senescence, ageing, inflammation, astrocyte, telomere, transport

## Abstract

**Introduction:**

A considerable body of recent evidence supports citrate transport as a major regulator of organismal lifespan and healthspan. Citrate accumulates outside senescent cells *in vitro* and *in vivo*. However, the detailed mechanism of senescent cell extracellular citrate (EC) accumulation is not clear.

**Methods:**

EC following various drug and cytokine treatments was measured in human fibroblast and keratinocyte conditioned medium by gas chromatography/mass spectroscopy and liquid chromatography/mass spectroscopy. Membrane transporters in similar human fibroblasts cultures were measured by western blotting and more extensively by reverse transcription and quantitative polymerase chain reaction (qPCR) in human fibroblasts, keratinocytes, myoblasts, adipocytes and astrocytes. Mouse tissues were tested for senescence markers and by qPCR, immunofluorescence and immunoFISH telomere associated foci (TAF) staining. Cytokine levels in conditioned medium were measured by the enzyme-linked immunosorbent assay and in mouse brain tissue and plasma samples using the V-PLEX proinflammatory panel 1 mouse kit.

**Results and Discussion:**

We show here that EC is partially mediated by a newly described plasma membrane citrate transporter *ANKH/SLC62A1* (progressive human ankylosis -*ANKH*) in senescent fibroblasts. Analogous to interleukin 6 (IL-6), EC and/or *ANKH* are regulated by telomere dysfunction, the p38 mitogen-activated kinase axis, transforming growth factor beta and p53, but in contrast not by steroids, sodium butyrate, or Ataxia Telangiectasia Mutated (ATM). ANKH was upregulated in other senescent cell types relevant to ageing but not keratinocytes. In contrast, EC and *ANKH* were inhibited by interleukin 1α (IL-1α) in dividing and senescent fibroblasts, accompanied by an increase in IL-6 secretion. Loss- and gain of function mutations of *ANKH/Ank* are associated with disease and interestingly, *Ank* is also downregulated in both aged mouse liver and brain tissues in parallel with increased senescence markers and several cytokines, suggesting that inflammatory cytokines could inhibit EC production *in vivo*. These data identify *ANKH/Ank* as a novel regulator of senescence-derived EC in both humans and mice.

## Graphical Abstract

**Figure:**
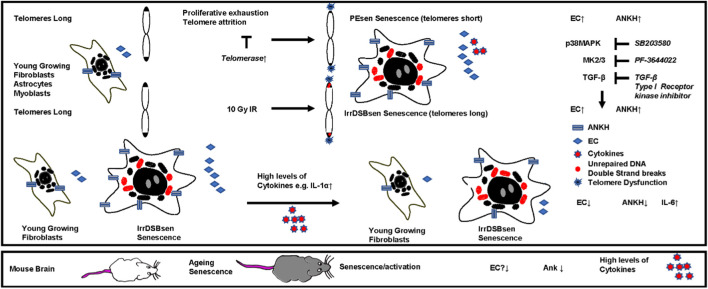

## 1 Introduction

Senescent cells are important to the progression of a wide variety of age-related conditions including Alzheimer’s disease, Parkinson’s disease, atherosclerosis, osteoporosis, osteoarthritis, frailty, type 2 diabetes, memory loss, hepatic steatosis, and cancer (Campisi et al., 2019). The deletion of senescent cells in mice slows ageing and ameliorates age-related conditions. However, our understanding of the mechanisms by which senescent cells contribute to these diseases, especially in human subjects, remains incomplete.

Citrate, a metabolite of the tricarboxylic acid cycle, can move between the cytoplasm and the extracellular space in both directions (Bhutia et al., 2017; Szeri et al., 2020) and accumulates in the body fluids of both ageing humans (Lin et al., 2021) (reviewed by Mycielska et al. (2022)) and mice (Varshavi et al., 2018; Yue et al., 2022), including cerebrospinal fluid (Lin et al., 2021) and urine (Varshavi et al., 2018). A considerable body of recent evidence supports citrate uptake and export as major regulators of organismal lifespan and healthspan (Birkenfeld et al., 2011; Branco et al., 2022; Fan et al., 2021a; Fan et al., 2021b; Willmes et al., 2021; Wu et al., 2024; Zahn et al., 2022), including their roles in cancer development (Drexler et al., 2021; Mycielska et al., 2018). However, whether citrate has detrimental or protective effects depends on factors such as diet, tissue, and species (Bhutia et al., 2017; Birkenfeld et al., 2011; Branco et al., 2022; Fan et al., 2021a; Fan et al., 2021b; Willmes et al., 2021; Zahn et al., 2022) and is normally tightly regulated (Jang et al., 2019). Therefore, it remains unclear whether citrate is beneficial or detrimental to health and in which context.

Our previous work has shown that extracellular citrate (EC) is upregulated following proliferative exhaustion (PEsen) or irreparable DNA double-strand break (IrrDSBsen)-induced fibroblast senescence (James et al., 2015) and is regulated by telomerase *in vitro* and in the human disease dyskeratosis congenita *in vivo* (James et al., 2023). A possible explanation for this increase in EC is that senescent fibroblasts (James et al., 2015) and melanocytes (Park et al., 2023), but not breast epithelial cells (Delfarah et al., 2019), shift their energy metabolism towards glycolysis as intracellular citrate inhibits phosphofructokinases 1 and 2, thereby suppressing glycolysis (Jenkins et al., 2011). Interestingly, inhibiting glycolysis may slow senescence *in vitro* (Park et al., 2023; Ma et al., 2023) and improve diabetic wound repair in mice (Ma et al., 2023). Consequently, citrate export and glycolysis may represent mechanisms for regulating senescent cell production and ageing, but the mechanism of EC production by senescent cells and its importance for healthy ageing remain unclear.

Recently, a plasma membrane transporter, progressive ankylosis protein homologue *ANKH/SLC62A1* (*ANKH*), previously known to transport pyrophosphate, was shown to export citrate and, to a lesser extent, malate and succinate (Szeri et al., 2020). However, our previous work found that senescent fibroblasts do not show elevated levels of inorganic phosphate or pyrophosphate (James et al., 2015). *ANKH*/*Ank* loss-of-function mutations lead to ankylosis due to the retention of pyrophosphate in the bone (Ho et al., 2000), and citrate accumulation in vascular smooth muscle cells leads to aortic aneurism (Wu et al., 2024). Furthermore, *ANKH* mutations have also been linked to chondrocalcinosis, arterial calcification, and arthritis (Pendleton et al., 2002). *Ank* knockout mice (Szeri et al., 2020) and humans with loss of *ANKH* function (Shin et al., 2014) have lower levels of plasma and urine citrate, and forced expression of *ANKH in vitro* increases extracellular citrate (Szeri et al., 2020). In addition, recent extensive genome-wide association screens revealed an association of the *ANKH* gene with a risk of developing Alzheimer’s disease (AD) (Bellenguez et al., 2022; Tesi et al., 2024), other forms of dementia (Katsumata et al., 2022), and type II diabetes (Nugent et al., 2020). Overall, ANKH appeared to be a good candidate for a role in citrate export in senescent cells, but little was known of its connection with senescence or its regulation by known senescence regulatory pathways.

We report that *ANKH* and EC are upregulated concomitantly in a variety of senescent cells and that both were regulated by some of the molecular pathways that regulate the senescence-associated secretory phenotype (SASP) proteins, but not all. Our results identify a potentially important role of *ANKH*/*Ank* in the regulation of EC in many types of senescent cells and, by inference, age-related diseases.

## 2 Materials and methods

### 2.1 Cell culture

#### 2.1.1 Cells

BJ cells at 16 mean population doublings (MPDs) were a generous gift from Professor Woodring Wright of Southwestern University, Dallas, Texas, United States, and were used between 22 and 25 MPDs. Later-passage BJ cells were obtained from the American Type Culture Collection and passaged until they became senescent (PEsen). Normal human oral fibroblast lines NHOF-1, NHOF-2, and NHOF-7 were derived from the explant cultures of normal oral mucosa and used at early passage between 18 and 22 MPDs, and NHOF-1 was serially passaged until PEsen, between 65 and 70 MPDs (James et al., 2015). IMR90 cells at early passage were used between 1.9 and 15 MPDs after receipt. Late-passage IMR90 cells were obtained from the American Type Culture Collection (Cat# CCL-186) at 28 MPDs and cultured until they reached PEsen at between 58 and 60 MPDs.

Cells were cultured in Dulbecco’s modified Eagle medium (DMEM, 4.5 g/L glucose, Lonza (Cat# BE12-604F/12, Slough, UK, and Thermo Fisher Scientific, Cat# 41966029) and supplemented with penicillin and streptomycin antibiotics (Life Technologies, Cat# 15070-063) to a concentration of 50 U/mL and L-glutamine (Life Technologies, Cat# 25030-081) to a concentration of 2 mM, containing 10% vol/vol foetal bovine serum (HyClone FetalClone II foetal bovine serum (Thermo Fisher Scientific, Cat# SH30066.02) at 37°C in an atmosphere of 10% CO_2_/90% air. A humid Eppendorf Galaxy S incubator was used. Flasks were kept at roughly 80% confluence, and the medium was replenished every 3–4 days. Once cells reached more than 80% confluence or were needed for an experiment, they were washed once with warm (37°C) PBS containing 0.02% weight/vol EDTA, then incubated for 5 min with PBS containing 0.1% weight/vol trypsin (Worthington crystalline, Lorne Laboratories, UK, Cat# TRL LS003703) and 0.01% weight/vol EDTA (ethylenediaminetetraacetic acid, Sigma-Aldrich, Gillingham, Dorset, UK, Cat# E51341mL/10 cm^2^ dish). Following cell detachment, trypsin was neutralised by the addition of serum containing media (3 mL media for every 1 mL trypsin solution), and cells were counted manually using a haemocytometer to enable the calculation of cumulative MPDs (cMPDs). MPDs were used throughout the study as a measure of chronological age and were calculated using the formula: MPDs = 3.32((log_10_cell number yield)−(log_10_cell number input) (Munro et al., 2001).

#### 2.1.2 Retroviral infection of BJ and NHOF-1 cells

pBABE retroviral vectors on the puromycin-resistant backbone expressing *TERT* and *TERT-HA* (Counter et al., 1998), the dominant-negative catalytically dead *TERT DNTERT* (Hahn et al., 1999), or the empty vector were obtained from Addgene Europe (Teddington, Middlesex, UK; Cat# 1771, 1772 and 1775, respectively). Retroviral vectors were transfected into Phoenix amphotropic 293T cells (Swift et al., 2001) to create infectious amphotropic retrovirus in 48 h. The conditioned medium containing the retrovirus was filtered using a 0.45 μm filter to remove Phoenix A producer cells and then incubated with sub-confluent late-passage NHOF-1 (45 MPDs) or BJ cells (65 MPDs) for a further 48 h without polybrene as this is not recommended for the infection of normal cells. The infected and mock-infected NHOF-1 and BJ cells were then trypsinised and replated at a density of 5 × 10^5^ cells per T75 flask and cultured for a further 48–72 h before adding 1 μg/mL of puromycin as a selecting agent. Puromycin was included in the medium until all the cells on the mock-infected plates had died, after which it was removed, as selection agents can reduce the replicative lifespan of normal cells (Morgan et al., 1987). Reintroducing selection before analysis did not change the expression of the *TERT* transgenes. Cells were expanded in control medium lacking puromycin, cryopreserved, and serially passaged when confluent at 1.5–3 x 10^3^ cells per cm^2^ until the control cells senesced. The medium was changed twice weekly.

#### 2.1.3 *Mycoplasma* testing

NHOF-1 was originally evaluated for *mycoplasma* using the MycoFluor *Mycoplasma* Detection Kit (Thermo Fisher, Cat #M7006) and found to be negative. Conditioned media from both BJ and NHOF-1 cell line panels were evaluated for *mycoplasma* using the Lonza MycoAlert™ *Mycoplasma* Detection Kit (Lonza, Cat# Lonza™ LT07-518) and were found to be negative.

#### 2.1.4 Astrocyte culture

Human astrocytes were obtained from ScienCell Research Laboratories (Cat# 1800) and cultured in the astrocyte growth medium (ScienCell Research Laboratories, AM, Cat. #1801) supplemented with 2% vol/vol foetal bovine serum (Cat. #0010), 1% vol/vol astrocyte growth supplement (AGS, Cat. #1852), and 1% vol/vol of the penicillin/streptomycin solution (Cat. #0503).

Upon thawing ampoules from liquid nitrogen or during sub-culturing, the astrocytes were plated onto poly-L-lysine-coated culture vessels. Vessels were pre-treated by adding 2 μg/cm^2^ (10 mL of sterile water to a T-75 flask and 15 μL of the poly-L-lysine stock solution (10 mg/mL, Cat. #0413) or the equivalent thereof) to the appropriate culture vessel. The vessels were incubated at 37^o^C overnight and washed twice with sterile double-distilled water before use. The cells were disaggregated as described above when approximately 50% confluent and replated at approximately 1 × 10^5^ cells per T25 flask before use in experiments. The cells were maintained in an atmosphere of 5% CO_2_/95% air.

The cMPDs and the states of senescence, PEsen and IrrDSBsen, were analysed as indicated above.

#### 2.1.5 Adipocyte culture

Human sub-cutaneous adipocytes were obtained from Cell Applications Inc. and cultured in pre-adipocyte growth medium (Cell Applications Inc., Cat# K811-500) supplemented with 5% vol/vol foetal bovine serum, 0.4% vol/vol endothelial cell growth supplement, 10 ng/mL recombinant epidermal growth factor, 1 μg/mL hydrocortisone, and 1% vol/vol and penicillin and streptomycin. The cells were disaggregated as described above when approximately 50% confluent and replated at approximately 1 × 10^5^ cells per T25 flask before use in experiments. The cells were maintained in an atmosphere of 5% CO_2_/95% air.

The cMPDs and the states of senescence, PEsen and IrrDSBsen, were analysed as indicated above.

#### 2.1.6 Induction of adipocyte differentiation

To induce differentiation, the cells were plated at 4.4 × 10^5^ per cm to reach confluence and maintained in the Cell Applications Adipocyte Differentiation Medium (Sigma Aldrich, Gillingham, Dorset, UK, Cat# 811D-250) for 15 days but were not subjected to starvation as this might have affected cellular senescence and citrate metabolism.

#### 2.1.7 Oil Red staining

Adipocytes and pre-adipocytes were stained with Oil Red O using a commercial kit from Biovision (Cat# K580-42), and the intensity of staining semi-quantified by measuring the absorbance. After staining with haematoxylin and washing with distilled water, the cells were washed an additional three times with 60% isopropanol (5 min each time with gentle rocking). The Oil Red O stain was then extracted with 100% isopropanol for 5 min with gentle rocking (250 µL per well); a measure of 80% of extraction volume was used for measurement. A well containing only 100% isopropanol was used as a background control, and its absorbance was subtracted from the sample reading. Absorbance was measured at 492 nm using a CLARIOstar plate reader (BMG LABTECH).

#### 2.1.8 Induction of senescence by ionising radiation (IrrDSBsen)

Fibroblasts, keratinocytes, astrocytes, and adipocytes were irradiated in suspension with 10 Gy of γ-rays from a Cs source at a dose rate of 1.4 Gy/min (James et al., 2015) or in later experiments with 10 Gy of X rays at a dose rate of 3.6 Gy/min to induce irreparable DNA double-strand breaks. The cells were maintained in culture for between 0 and 20 days before analysis. Where indicated, keratinocytes were *in situ* irradiated with γ-rays following the removal of the 3T3 feeder cells by EDTA treatment and vigorous pipetting, as described (Karen-Ng et al., 2022). 3T3 feeder cells were irradiated with 60 Gy of either γ- or X-rays, as indicated above (Karen-Ng et al., 2022).

### 2.2 Experimental design and drug treatment

Cells were seeded in a manner that ensured comparable densities between senescent and growing controls; however, to induce short-term confluence, higher numbers of cells were seeded to promote a short-term reversible growth arrest. Where indicated, the numbers of senescent cells seeded was adjusted to match the level of confluence.

Cells were seeded for 3–5 days prior to changing the medium for 16 h before lysis or collection of conditioned media. Except for the long-term NaB experiments, all drugs, growth factors, cytokines, and inhibitors were added for the indicated time and during the final 16 h prior to analysis. Controls consisted of either culture medium with no additives or vehicle controls (ethanol or DMSO at 0.1% vol/vol).

For shRNA induction, cells were cultured in the indicated concentration of doxycycline hyclate (DOX; Thermo Scientific, Cat# ICN19895510) for 1 week prior to setting up the experiment and throughout its duration. Non-induced and DOX-induced cells expressing non-targeting vectors served as controls.

### 2.3 Source of other chemicals

#### 2.3.1 Cytokines and steroids

TGF beta 1 (R&D Systems, Cat# 240B-002); recombinant human interleukin 1 alpha (IL-1α/IL-1F1) (R&D Systems, Cat#: 200-LA); corticosterone (Sigma-Aldrich, Gillingham, Dorset, UK, Cat# 27840); and hydrocortisone hemisuccinate (cortisol) (Sigma-Aldrich, Gillingham, Dorset, UK, Cat# H2270).

#### 2.3.2 Pathway inhibitors

KU55933 (R&D Systems, Cat# 3544/10); SB203580 (Tocris Chemical Company, Cat# 1202); PF-3644022 (Tocris Chemical Company, Cat# 4279); TGFBR1 kinase inhibitor 1 LY-364947 (Calbiochem, Cat# 616451); and TGFBR1 kinase inhibitor 2 (Calbiochem, Cat# 616452).

#### 2.3.3 Miscellaneous chemicals

2′, 7′-dichlorodihydrofluorescein diacetate (H2DCFDA) (Calbiochem, Cat# 287810).

Tert-butylhydroperoxide (Luperox TBHP solution, Sigma-Aldrich, Gillingham, Dorset, UK, Cat# 458139, lot# BCBG4467V).

N-tert-butyl-a-phenylnitrone (Sigma, Gillingham, Dorset, UK, Cat# B7263).

Puromycin (Sigma, Gillingham, Dorset, UK, Cat# P8833).

Sodium butyrate (Sigma, Gillingham, Dorset, UK, Cat# B5887).

### 2.4 Characterisation of senescent cells

The senescence status of the cells was confirmed by assessing senescence-associated beta galactosidase (SA-βGal) activity using commercial kits—initially the Biovision Kit (Cat# K-320-250) and subsequently the Generon Senescence Activity Assay Kit (Cat# KA6035); SA-βGal activity was re-evaluated at the time each experiment was conducted. In brief, the kits were brought to room temperature for 20 min. Following this, the cells were washed twice with calcium and magnesium-free phosphate-buffered saline (PBS), fixed for 10 min, and washed an additional two times before adding the SA-βGal reagent. The plates were then sealed with parafilm to prevent dehydration, covered in foil to protect from light, and incubated at 37^o^C in a hot room or hotbox in an atmosphere of air (0.03% CO_2_). Late-passage IMR90 cells were used as positive controls.

### 2.5 Western blotting

The following antibodies were used: pmCiC and mCiC (SLC25A1) rabbit polyclonal antibodies (Mazurek et al., 2010), custom-made by GenScript Inc.; ANKH (rabbit, VivaSystems, Cat# OAAB06341), MCT1 (rabbit, Santa Cruz, Cat# sc-365501), MCT4 (goat, Santa Cruz, Cat# sc-14930), Actin C-4 (mouse, Santa Cruz Cat# sc-47778), and ASCT2 (rabbit, Cell Signaling, Cat# 5345).

In brief, cell extraction was performed to isolate proteins from confluent cultures. Cells were washed with PBS and immediately extracted with 2× sodium dodecyl sulphate (SDS) Laemmli sample buffer (0.5 m Tris‐Cl, pH 6.8; 4% SDS; 20% glycerol), with 10% (v/v) 2‐mercaptoethanol added after the protein assay). DC Protein Assay (Bio‐Rad, Hercules, CA, USA) was used to determine the protein concentration for each sample. Approximately 25 μg (10–30 µg) of protein was prepared with 5 µL Laemmli buffer, centrifuged at 15,000 rpm (20,000 g), boiled at 95°C for 5 min, spun down, centrifuged again, and stored on ice.

The proteins were separated on 10% SDS polyacrylamide gels at 100 V for 90–150 min and transferred to nitrocellulose filters (Whatman Merck, Darmstadt, Germany) in SDS running buffer and methanol at 100 V for 50–60 min. After blotting, the membrane was placed in 5% w/v-milk powder in Tris-buffered saline containing 10% w/v SDS, glycine, 0.1% vol/vol Tween 20 (Sigma-Aldrich, Poole, Dorset, UK, Cat# P2287) (TBS-T) on shaker for 1 h. Following blocking, the primary antibodies were diluted 1:1,000 in TBS-T and 5% w/v/milk powder and incubated at 4^o^C overnight. The following day, the membranes were washed twice for 15 min each in TBS-T with agitation. The membranes were then incubated with the appropriate secondary antibody at a dilution of 1:2,500 in TBS-T and 5% w/v/milk powder for 1 h at room temperature. The membranes were washed twice for 15 min each in TBS-T with agitation before incubating with freshly prepared ECL for 2 min, developing with X-ray film developer in the dark, and marking the molecular weight markers on the film. The membranes were washed with TBS-T as described above before being reprobed with the loading control.

### 2.6 Collection of conditioned medium

Conditioned medium was collected after 24 h from the cells and centrifuged at 800 x g for 2 min. The supernatant was removed and centrifuged again at 15,000 rpm for 2 min, and the final supernatant was snap-frozen in an ethanol–dry ice bath for 15 min before storage at −80°C. Unconditioned medium was also prepared identically.

### 2.7 Targeted measurement of extracellular citrate by gas chromatography/mass spectrometry

All chemicals and standards were obtained from Supelco (Sigma-Aldrich, Gillingham, Dorset). Deuterated citrate (Citrate-d_4_, Cat# 485438) was added to each sample to a final concentration of 0.1 mM as an internal standard. Metabolites were then extracted using cold methanol before being dried under vacuum desiccation. The samples were re-suspended in anhydrous pyridine containing the derivatisation agent methoxyamine hydrochloride (Cat# 69479), followed by N-methyl-N-trimethylsilyltrifluoroacetamide (Cat# 394866) with 1% 2,2,2-trifluoro-N-methyl-N-(trimethylsilyl)-acetamide (Cat# 111805) and chlorotrimethylsilane (MSTFA + 1%TMCS, Cat# 92361). Gas chromatography/mass spectrometry (GC-MS) was performed in pulsed split-less mode on a Hewlett Packard HP6890 series GC system equipped with an Agilent 6890 series injector and a 30 m × 250 µm capillary column (Agilent, model number 19091s-433HP5MS), operated at a flow rate of 1 mL/min, and coupled to a Hewlett Packard 5973 mass selective detector. Acquisition was conducted in selective ion monitoring mode, with the m/z values 273, 347, 375, and 465 for citrate and 276, 350, 378 and 469 for citrate-d_4_. The dwell time for all these ions was 50 ms.

The values were normalised against cell numbers, as described in our previous publications (James et al., 2015; James et al., 2018).

### 2.8 Targeted measurement of extracellular citrate by liquid chromatography/mass spectroscopy

We measured citrate using a targeted liquid chromatography/tandem mass spectroscopy (LC-MS-MS) method, incorporating a mixed-mode UPLC chromatographic separation step that enabled retention and discrimination of organic acids, including excellent resolution of citrate from isocitrate. The media samples were diluted 10-fold with cold methanol and then diluted again 5-fold with cold methanol containing an internal standard (1 µg/mL L-phenyl-d_5_-alanine (Sigma-Aldrich, Gillingham, Cat# 615870). The mixture was vortexed at 6°C for 20 min and then centrifuged (17,000 g, 10 min, 4°C). A measure of 490 μL aliquots of the supernatant was dried under reduced pressure and stored at −80°C until analysis.

We then re-suspended the dried samples in 50 µL of water containing the stable-isotope-labelled (SIL) standard ^13^C_3_-citrate (Cambridge Isotope Laboratories, CLM-9876) and measured citrate essentially as described by Smith et al. (2021) with modification of the LC gradient. Specifically, the initial binary elution gradient was as follows: 0% B at 0.0 min; 0% B at 0.1 min; 25% B at 8.1 min; 95% B 11.1 min; held at 95% B until 12.1 min; reduced to 0% B at 13 min; and maintained at 0% B until 15 min. The separation was performed using an ACQUITY Premier CSH Phenyl-Hexyl Column (2.1 × 100 mm, 1.7 μm). The analysis was performed on a XEVO TQ-S (Waters, UK) using negative ionisation mode, with the following settings: capillary voltage, 1.5 kV; source offset, 50 V; desolvation temperature, 500°C; source temperature, 150°C; desolvation gas flow, 1000 L/h; cone gas flow, 150 L/h; nebuliser gas, 7.0 bar; and collision gas, 0.15 mL/min. The transitions used in MS were detailed as follows: citrate Q1 191.0197 *m/z* → Q3 111.1000 *m/z*, collision energy 12 V; Q1 191.0197 *m/z* → Q3 87.1000 m/z, collision energy 18 V; and ^13^C_3_ citrate Q1 194.0197 *m/z* → Q3 113.1000 *m/z*, collision energy 12 V; and Q1 191.0197 m/z → Q3 87.1000 m/z, collision energy 18 V. All the transitions were set with a dwell time of 0.008 s and a cone voltage of 45 V.

We quantitated citrate against an authentic standard (Supelco, Sigma Aldrich, Gillingham, Dorset, Cat# C6272) using a 6-point calibration curve (from 0.025 to 1 mg/L, run immediately before and after the samples), along with the SIL ratio. The results were checked against both water and method blanks.

### 2.9 Reverse transcription and quantitative PCR

Cultured cells were lysed in lysis buffer, and mRNA extraction was performed as per the manufacturer’s instructions using the Dynabeads mRNA DIRECT Kit (Thermo Fisher, UK, Cat# 61012). The purified mRNA sample was then combined with qPCRBIO SyGreen 1-Step Go (PCR Biosystems, Cat# PB25.31-12) master mix and qPCR primers for one-step relative quantification of target genes and two reference genes using a 384-well format LightCycler 480 qPCR system (Roche), following our previously published protocols (Teh et al., 2013; Gemenetzidis et al., 2009; Teh et al., 2010; Waseem et al., 2010), which are MIQE compliant (Bustin et al., 2009). In brief, thermocycling begins with 45°C for 10 min for reverse transcription, followed by 95°C for 30 s, prior to 45 cycles of amplification at 95°C for 5 s, 60°C for 5 s, 72°C for 5 s, and 78°C for 1 s (data acquisition). A “touch-down” annealing temperature intervention (66°C starting temperature with a stepwise reduction of 0.6°C/cycle; 8 cycles) was introduced prior to the amplification step to maximise primer specificity. Melting analysis (95°C for 30 s, 75°C for 30 s, and 75ºC–99°C at a ramp rate of 0.57°C/s) was performed at the end of qPCR amplification to validate single-product amplification in each well. Relative quantification of mRNA transcripts was calculated based on an objective method using the second derivative maximum algorithm (Zhao and Fernald, 2005) (Roche). All target genes were normalised to two reference genes (HPRT1 and B2M), validated previously (Gemenetzidis et al., 2009) to be amongst the most stable reference genes across a wide variety of primary human epithelial cells, dysplastic, and squamous carcinoma cell lines, using the geNorm algorithm (Vandesompele et al., 2002). Relative expression data were then exported into Microsoft Excel for statistical data analysis. No template controls (NTCs) were prepared by omitting the tissue sample during RNA purification, and eluates were used as NTCs for each qPCR run. The qPCR was carried out for the mouse *Ank* gene in the same way as for the human *ANKH* gene.

#### 2.9.1 Human qPCR primer sequences

The custom oligonucleotides were obtained from Thermo Fisher Scientific, Paisley, Cat# 10336022.

**Table:** 

Gene	Sequence 5′-3′	Product size (bp)
*B2M-F*	AAG​TGG​GAT​CGA​GAC​ATG​TAA​G	70
*B2M-R*	GGA​ATT​CAT​CCA​ATC​CAA​ATG​CG	
*HPRT1-F*	TGA​CAC​TGG​CAA​AAC​AAT​GCA	93
*HPRT1-R*	GGT​CCT​TTT​CAC​CAG​CAA​GCT	
*ANKH-F*	ACC​AAA​GCC​GTC​CTG​TGT​A	112
*ANKH-R*	CCA​CAT​GGT​GCA​GTT​TAT​TGA	
*ANKH-2F*	CGG​CCT​ATT​GTC​AAC​CTC​TTT	78
*ANKH-2R*	TGTCAAAATCGCCACTGC	
*p16-F*	TGC​CCA​ACG​CAC​CGA​ATA​G	176
*p16-R*	CAC​CAG​CGT​GTC​CAG​GAA​G	
*p53-F*	AGG​CCT​TGG​AAC​TCA​AGG​AT	85
*p53-R*	CCC​TTT​TTG​GAC​TTC​AGG​TG	
*p21-F*	TCA​CTG​TCT​TGT​ACC​CTT​GTG​C	127
*p21-R*	GGC​GTT​TGG​AGT​GGT​AGA​AA	
*IVL-F*	TGC​CTG​AGC​AAG​AAT​GTG​AG	83
*IVL-R*	TTC​CTC​ATG​CTG​TTC​CCA​GT	
*K10-F*	AAA​CCA​TCG​ATG​ACC​TTA​AAA​ATC	134
*K10-R*	GCG​CAG​AGC​TAC​CTC​ATT​CT	
*IL1A-F*	TGC​CCA​AGA​TGA​AGA​CCA​ACC	70
*IL1A-R*	ACT​ACC​TGT​GAT​GGT​TTT​GGG​T	
*IL1B-F*	CCT​CCA​GGG​ACA​GGA​TAT​GG	92
*IL1B-R*	CCA​AGG​CCA​CAG​GTA​TTT​TGT​C	
*IL6-F*	AAG​TCC​TGA​TCC​AGT​TCC​TGC	77
*IL6-R*	GGC​ATT​TGT​GGT​TGG​GTC​AG	
*CXCL1-F*	AAC​CGA​AGT​CAT​AGC​CAC​ACT	117
*CXCL1-R*	TCT​GGT​CAG​TTG​GAT​TTG​TCA​CT	
*C3-F*	GGG​CGT​GTT​CGT​GCT​GAA​TA	78
*C3-R*	CCG​ATG​TCT​GCC​TTC​TCC​AC	
*B2m-F*	TAC​GCC​TGC​AGA​GTT​AAG​CA	71
*B2m-R*	GAT​CAC​ATG​TCT​CGA​TCC​CAG​T	
*Hprt1-F*	GGG​GGA​CAT​AAA​AGT​TAT​TGG​TGG	70
*Hprt1-R*	TTC​AAC​AAT​CAA​GAC​ATT​CTT​TCC​A	
*Ank-F*	TCA​CCA​CAT​AGC​CAT​CGA​C	73
*Ank-R*	ACT​GCA​TCC​TCC​TGA​CTG​C	
*Ank-2F2*	CTG​CTG​CTA​CAG​AGG​CAG​TG	215
*Ank-2R2*	GAC​AAA​ACA​GAG​CGT​CAG​CGA	
*Ank-2F3*	AAC​TGG​CGA​ACA​CAA​GCA​AC	92
*Ank-2R3*	GAC​AAA​ACA​GAG​CGT​CAG​CG	

### 2.10 Knockdown of target genes by conditionally expressed short hairpin RNA (shRNA) constructs

We used pre-prepared lentiviral particles from Horizon Discovery (Cambridge, UK; SMARTvector Inducible Lentiviral shRNAs) to infect the indicted human cells.

First, we identified the promoter that provided the most effective induction of shRNA, indicated by either green fluorescent protein (GFP) or red fluorescent protein (RFP). This was achieved using the SMARTchoice Inducible Non-Targeting Control 4-Pack (Cat# VSC6847), following the manufacturer’s instructions. Based on these pilot experiments, we determined that the mouse cytomegalovirus promoter yielded the strongest induction and, therefore, proceeded with constructs using this promoter backbone.

The indicated cells were infected with lentiviral particles in transduction medium (serum-free DMEM high glucose without L-Glut or sodium pyruvate) at a multiplicity of infection of 0.3 virus particles per cell. The following day, the cells were changed into regular culture medium, and they were trypsinised and plated at 1 × 10^5^ cells per T75 flask on day 3. After a further incubation time of 4 days, 1 μg/mL of puromycin was added until all the mock-infected cells were dead, and then, puromycin was removed prior to the expansion of cell populations and cryopreservation. We used this protocol because culturing normal cells in selection agents can reduce replicative lifespan.

The dose of DOX (Thermo Scientific, Cat# ICN19895510) was optimised by measuring GFP or RFP fluorescence following 4 days of induction. The toxicity of DOX was estimated in parallel by the addition of MTT to the cells for 1 h in serum-free DMEM, followed by dissolving the cells in dimethyl sulphoxide (DMSO), and absorbance was measured at a wavelength of 570 nm. The level of knockdown by each shRNA was monitored by qPCR, as indicated below. To achieve maximum knockdown efficiency, the induced cells were enriched by flow sorting to exclude low-expressing cells, selecting only those with high GFP or RFP expression after induction with DOX, using non-induced cells as the background. We chose three shRNAs, validated by Horizon Discovery, targeting both *ANKH* and *TP53*.

#### 2.10.1 shRNA targeting sequences

**Table:** 

Target	Fluorochrome	Targeting sequence
Non-targeting	Mouse CMV turbo GFP	Cat# VSC6570
Non-targeting	Mouse CMV turbo RFP	Cat# VSC6571
ANKH#1002	Mouse CMV turbo GFP	Cat# V3SH7669-227064850 GGCACCAAGAACCGGATCA
ANKH#2500	Mouse CMV turbo GFP	Cat# V3SH7669-230823352 TGCTCCCCGAAGTCGATGG
ANKH#5474	Mouse CMV turbo GFP	Cat# V3SH7669-228017824 TTTGAAGTCACTCATGGGA
TP53#1	Mouse CMV turbo RFP	Cat# V3SH7669-227164253 CACACGCAAATTTCCTTCC
TP53#2	Mouse CMV turbo RFP	Cat# V3SH7669-225233423 AGGCCCTTCTGTCTTGAAC
TP53#3	Mouse CMV turbo RFP	Cat# V3SH7669-225995888 TATTTCATTAACCCTCACA

### 2.11 Enzyme-linked immunosorbent assay

To measure IL-6 in conditioned media, a sandwich enzyme-linked immunosorbent assay (ELISA) method (Quantikine^®^ ELISA Immunoassay, R&D Systems, Abingdon, UK for IL-6 Cat# D6050) was employed, following the manufacturer’s protocol. The detection limit for IL-6 was 3.13-300 pg/mL.

### 2.12 Mouse experiments

The details and characterisation of the mouse liver and kidney tissues have been previously published (Rapisarda et al., 2017).

#### 2.12.1 Animals for aged brain analysis

The available strain for studying the role of ageing was C57BL/6, adult (8–10 weeks old) and aged (17 months old) male C57BL/6 mice, weighing 20–24 g at the start of the study (Janvier Laboratory, France). The Animal Welfare and Ethical Review Body, at Queen Mary University of London and the United Kingdom Home Office, following the EU Directive 2010/63/EU, approved all animal procedures.

#### 2.12.2 Animal culling and sample collection

To collect tissue, animals were deeply anaesthetised using sodium pentobarbital (50 mg/kg), administered intraperitoneally (i.p.) in a volume of 0.5 mL/kg. Brain tissue and blood samples were collected from all animals. Animals were either perfused with PBS, followed by 4% paraformaldehyde (PFA) for tissue analysis by immunostaining, or with PBS only for the cytokine analysis that required fresh frozen tissue. Blood samples were collected in lithium heparin tubes and then centrifuged at 10,000 g for plasma separation. Plasma samples were stored at −80°C for future analysis.

#### 2.12.3 Immunostaining

All tissue staining was performed on 8 μm coronal brain sections cut between bregma −1.9 mm and −2.1 mm. Sections were deparaffinised in two washes of xylene (10 min each), then hydrated in a series of ethanol (EtOH) baths (100% EtOH, 100% EtOH, 90% EtOH, and 70% EtOH, 5 min each), and then washed twice in PBS (5 min each). Then, sections were treated with a heated antigen retrieval solution containing 10 mM citric buffer (pH 6.0) for 10 min in a microwave. After that, sections were blocked with 8% bovine serum albumin (BSA), 0.5% Tween-20, and 0.1% Triton X-100 in PBS overnight in a humidified chamber at 4°C. The following day, sections were washed twice in 0.5% Tween-20 and 0.1% Triton X-100 in PBS (PBS-TT) and then incubated with primary antibodies (2.12.4) overnight in a humidified chamber at 4°C. Markers were visualised using secondary goat-raised antibodies, which were labelled with either Alexa 488 or Alexa 555 (Invitrogen, United States; 1:250), and nuclei were visualised using Hoechst 33342 stain (Tocris Bioscience, United Kingdom; 1:500). Slides were mounted and cover-slipped using VECTASHIELD fluorescent mounting medium (Vector Laboratories, United States).

#### 2.12.4 Antibodies used

**Table:** 

Marker	Antibody full name	Species	Company	Product ID	Dilution
Anti-p53	Anti-tumour protein p53	Rabbit	Insight Biotechnology, United States	GTX102965	1:150
Anti-p21^Waf^	Anti-cyclin-dependent kinase inhibitor 1	Rabbit	Invitrogen, United States	14-6715-81	1:150
Anti-p16^Ink4A^	Anti-cyclin-dependent kinase inhibitor 2A	Rat	Abcam, United Kingdom	ab241543	1:150
Anti-p19^Arf^	Anti-ARF tumour suppressor	Rat	Novus Biological, United States	NB200-169	1:150
Anti-γH2AX	Anti-phosphorylated H2A histone family member X	Mouse	Merck, KGaA, Germany	05-636	1:500
Anti-53BP1	Anti-tumour suppressor p53-binding protein 1	Rabbit	Cambridge Bioscience, United Kingdom	A300-273A	1:1600

#### 2.12.5 ImmunoFISH telomere associated foci staining

ImmunoFISH staining was used to detect senescent cells. First, sections were deparaffinised in two washes of xylene (10 min each), followed by rehydration through a graded ethanol series (100% EtOH, 100% EtOH, 90% EtOH, and 70% EtOH, 5 min each), and then rinsed in two changes of PBS (5 min each). After that, sections were treated with ice-cold (−20°C) 70% ethanol for 20 min and then washed three times in PBS for 15 min. Next, sections were blocked with 8% BSA (Sigma-Aldrich, Saint Louis, United States), 0.5% Tween-20 (Sigma-Aldrich, Poole Dorset, UK, Cat# p2287), and 0.1% Triton X-100 (Sigma-Aldrich, Poole Dorset, UK Cat# T8787) in PBS overnight at 4°C in a humidified chamber. The following day, sections were washed with PBS-TT for 5 min; subsequently, one or two primary antibodies were added and then left overnight at 4°C in a humidified chamber. One of them was an anti-phospho-histone γH2A.X antibody (Cell Signaling Technology, Danvers, United States; 1:250), and the second was anti-NeuN (Merck Millipore, Burlington, United States; 1:250). On day 3, sections were washed in PBS-TT for 5 min twice and then incubated with the following secondary antibodies: biotinylated anti-rabbit IgG (Vector Laboratories, Burlingame, United States; 1:250) and Alexa Fluor 647 (Far red; Invitrogen, Carlsbad, United States; 1:250) for 1 h in a humidified chamber. After that, sections were washed in PBS-TT for 5 min three times and then incubated with DSC-fluorescein (Vector Laboratories, Peterborough, UK, 1:500 in PBS) for 20 min. Later, sections were washed twice with PBS-TT, twice with PBS for 5 min, and then treated with 4% PFA in PBS for 20 min. Next, sections were washed 3 times with PBS for 5 min, followed by a dehydrating step using an ice-cold gradient of ethanol (70% EtOH, 90% EtOH, and 100% EtOH, 3 min each). Sections were air-dried and then treated with a hybridisation mix, which contained 0.5 μg/mL (C3TA2)3-Cy3-labelled peptide nucleic acid (PNA) telomeric probe, 70% formamide, 12 mM Tris-HCl (pH 7.4), 5 mM KCl, 1 mM MgCl_2_, 0.001% Triton X-100, and 2.5 mg/mL acetylated BSA (Sigma-Aldrich, St. Louis Missouri, United States, Cat# B2518). Sections were coverslipped and then incubated in an oven (82°C) for 10 min. The hybridisation process was continued in a humidified chamber for 2 hours. Finally, sections were washed in 70% formamide/2x SSC for 10 min, twice in 2x SSC for 10 min, twice in PBS for 10 min, and then another change of PBS for 30 min. Slides were mounted using VECTASHIELD fluorescent mounting medium with 4′,6-diamidino-2-phenylindole **(**DAPI) (Vector Laboratories, Burlingame, United States).

#### 2.12.6 Quantification of IF and TAF signals

Four coronal sections were used per animal for each antibody staining; the sections were situated along the rostral–caudal axis, with a spacing of 0.1 mm between them. Images were captured at x 40 magnification using the IN Cell Analyser 2200 (INCA2200) System (Cytiva, Marlborough, United States) for the markers: 8-oxo-dG, p53, p19^ARF^, p21^WAF^, and p16^INK4A^. For the γH2AX.53BP1 signal overlap and telomere-associated foci (TAF) staining, fields used for quantification were captured at x 60 magnification using the IN Cell Analyser 6000 (INCA6000) Confocal System (Cytiva, Marlborough, United States). A minimum number of 200 fields of view (FoV) were captured per animal across all four sections, with total nuclei counts of at least 3 × 10^4^ nuclei.

All quantitative analyses were conducted using the IN Cell Developer Toolbox v1.9.2 (Cytiva, Marlborough, United States); and custom protocols were developed for each marker evaluated, using the build-in parameter selection tools. A cell was considered positive for a marker when the fluorescent signal co-localised with a nucleus by at least a 95% overlap. Results were expressed as a percentage of the total nuclei detected.

### 2.13 Cytokine analysis

#### 2.13.1 Brain samples

Snap-frozen brain tissue was used for cytokine analysis. A measure of 25 mg tissue was used from each sample, and the tissue was cut into small pieces on ice. The tissue fragments were suspended in lysate buffer (RIPA buffer containing protease/phosphatase inhibitors) and then crushed with a Pellet Pestle^®^ (Sigma-Aldrich, United Kingdom). After that, samples were placed on rotating wheels and incubated for 20 min at 4°C. Next, samples were sonicated 3 times, 15–20 s each, followed by centrifugation for 20 min at 10,000 g (4°C). Then, the supernatant was collected from each sample and used for the bicinchoninic acid (BCA) assay to measure protein concentration, followed by the measurement of cytokine concentrations.

For the BCA assay, the Pierce BCA Protein Assay Kit (Invitrogen, Life Technologies Ltd, United Kingdom) was used to measure the protein concentration extracted from brain tissue, following the manufacturer’s instructions. Samples were diluted with the supplied MSD solution, Diluent 41, to reach a concentration of 2.5 mg/μL, as recommended.

#### 2.13.2 Plasma samples

Thawed plasma samples were diluted in a 1:3 ratio with the MSD supplied solution, Diluent 41, before being used in the assay.

Running the experiment and data processing

Cytokine levels in brain tissue and plasma samples were measured using the V-PLEX Proinflammatory Panel 1 Mouse Kit (Cat. No. K15048D-1; Meso Scale Diagnostics (MSD), United States), following the manufacturer’s. Prepared assay plates were analysed using MESO QuickPlex SQ 120 MM (MSD, United States).

### 2.14 Statistical methods

Human and mouse data, where the numbers of repeats were the same, were analysed using the Student’s unpaired t-test. Datasets containing more than one variable were also analysed using one-way or ordinary two-way ANOVA to confirm the results of the t-test. In scenarios where the repeat numbers were uneven, human data were analysed using the Welch’s T-test and the mouse data were analysed using the Mann–Whitney test. The significance level was set at p < 0.05. The significance of the mouse data was additionally analysed using the Cohen d test, with a cutoff for significance set at 1.

## 3 Results

### 3.1 Identification of *ANKH* as a candidate citrate exporter in human cellular senescence

**FIGURE 1:**
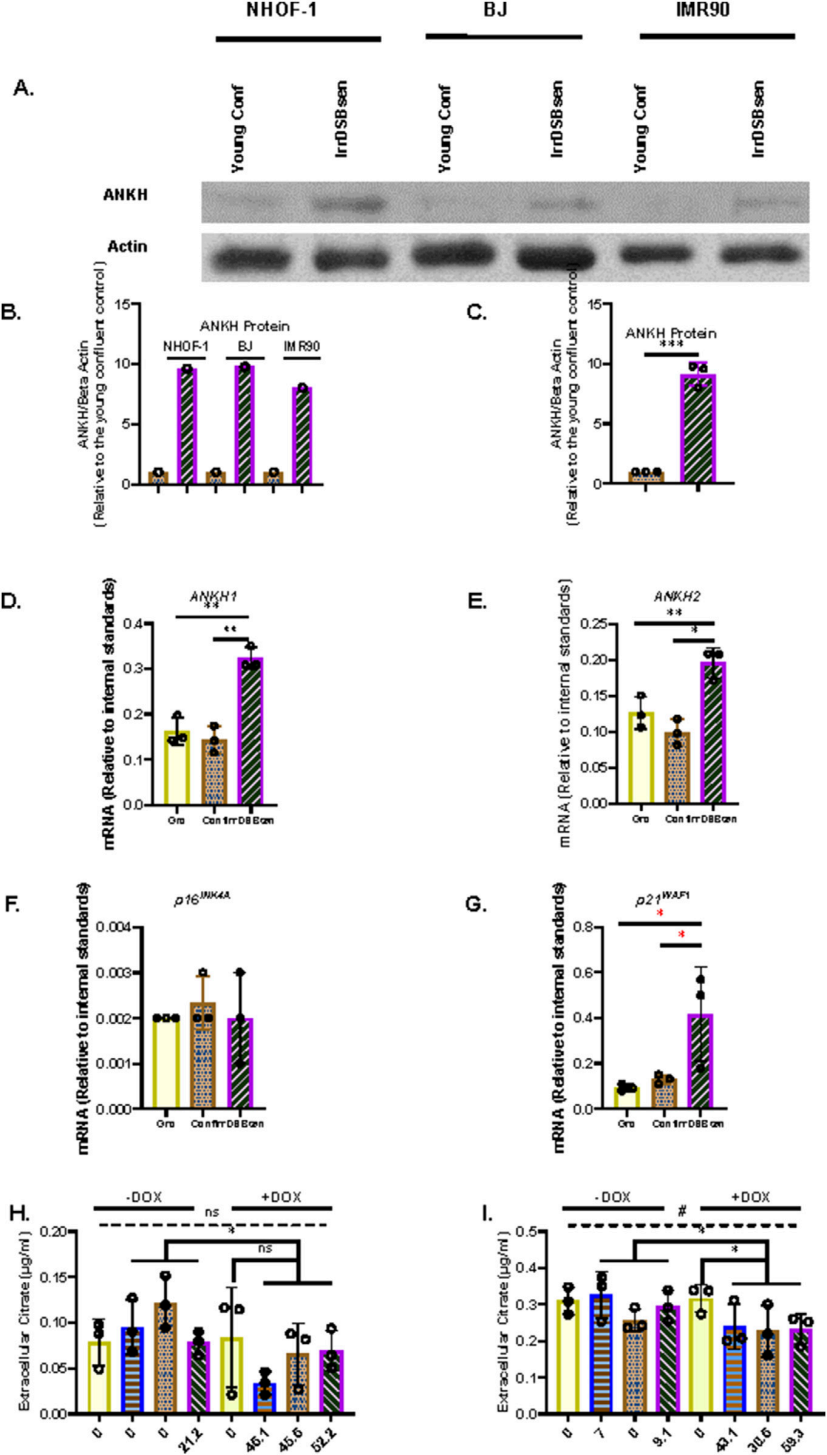
*ANKH* protein and transcript are upregulated in IrrDSBsen human fibroblasts. **(A)** Western blot of the *ANKH* protein in three lines of quiescent confluent (Conf) young human fibroblasts and the same lines induced to senesce by ionising radiation (IrrDSBsen). Beta action was used as a loading control. **(B)** ImageJ quantification of the blot in **(A)**. The levels of the ANKH signal versus beta actin in all conditions after background subtraction. The full-length blots are shown in Supplementary Figure S2. **(C)** Average of the values in **(B)**. **(D)** Expression of ANKH mRNA in growing confluent and IrrDSBsen NHOF-1 cells using primer set 1. **(E)**. This is similar to **(D)** but shows the results of primer set 2 in the same experiments. **(F)** This is similar to **(D)** but shows the expression of the senescence marker p16INK4A in the same experiments. **(G)** This is similar to **(D)** but shows the expression of the senescence marker p21WAF1 in the same experiments. Yellow plain bars, growing cells; orange stippled bars, confluent cells; purple hatched bars, IrrDSBsen cells. **(H)** Effect of inducible *ANKH* shRNA vectors and their non-targeting (NT) controls with and without the doxycycline inducer (DOX) on growing NHOF-1 cells. The numbers below each bar represent the level of *ANKH* mRNA knockdown. Yellow plain bars, NT control; blue horizontal striped bars, shRNA#1002; orange stippled bars, shRNA #2500; purple left-right diagonally striped bars, shRNA #5474. **(I)** Effect of inducible *ANKH* shRNA vectors and their NT controls with and without the doxycycline inducer (DOX) on IrrDSBsen NHOF-1 cells. The numbers below each bar represent the level of *ANKH* mRNA knockdown. The symbols are the same as for **(H)** (see also Supplementary Figure S3). In all experiments, *, P < 0.05; **, P < 0.01; ***, P < 0.001; *P > 0.05; <0.1; ns, not significant. Data in **(H,I)** were also analysed by one-way ANOVA (dashed line) #, P < 0.05; ns, not significant. The results are presented as the averages +/- standard deviation. N = 3. The experiments in **(A,C,D,G, H,I)** were performed at different times but using identical cell culture protocols and reagents.

Recently, *ANKH* was identified as a plasma membrane exporter of citrate and, to a lesser extent, malate (Szeri et al., 2020)—two metabolites that we found to accumulate in the conditioned medium of both PEsen and IrrDSBsen senescent fibroblasts (James et al., 2015). Hence, we screened three fibroblast lines (NHOF-1, BJ, and IMR90) for the expression of several plasma membrane transporters, including ANKH, in both confluent young (cell cycle arrested) and IrrDSBsen fibroblasts. The mitochondria/cytoplasm citrate exporter SLC25A1 (CiC), but not its plasma membrane form (pmCiC), was consistently upregulated in IrrDSBsen cells (Supplementary Figure S1). The same cells also showed an increase in *ANKH* expression in all three IrrDSBsen cultures (Figure 1A)—an increase of between 8- and 9.6-fold relative to the confluent young quiescent controls (Figures 1B,C). Other plasma membrane transporters, the monocarboxylate transporters MCT1 (Supplementary Figure S1) and MCT4 (Supplementary Figure S1), showed either no upregulation (MCT1) or undetectable expression (MCT4); the neutral amino acid transporter ASCT2 was downregulated in all three IrrDSBsen lines (Supplementary Figure S1). All the full-length blots are shown in Supplementary Figure S2. We next examined whether *ANKH* expression was upregulated in senescent fibroblasts at the mRNA level. In IrrDSBsen NHOF-1 cells, this was the case, as assessed by two *ANKH* primer sets (Figures 1D,E). P16^INK4A^ was not generally elevated in NHOF-1 cells upon senescence (Figure 1F), but another senescence effector, p21^WAF^, showed a strong trend for elevation in parallel to *ANKH* (Figure 1G).

### 3.2 The conditional knockdown of *ANKH* mRNA reduces EC in IrrDSBsen human fibroblasts

To evaluate whether *ANKH* was a mediator of EC in senescent fibroblasts, we transduced NHOF-1 fibroblasts with three commercially validated inducible shRNA vectors targeted against *ANKH* and a non-targeting (NT) control. We then measured the extent of *ANKH* mRNA knockdown and the effect on EC. The results confirmed our previously published data that EC was four-fold higher in the IrrDSBsen fibroblast conditioned medium than in the growing controls. Inducible shRNA knockdown of *ANKH* resulted in a reduction of *ANKH* mRNA expression by 46% to 52% in growing NHOF-1 cells following induction by doxycycline (DOX; P = 0.001 to P = 0.02; Supplementary Figure S3). However, this knockdown did not significantly reduce EC levels compared to the NT vector following induction by DOX (P = 0.47 using the Welch’s T-test; Figure 1H).This lack of significance is due to the very low levels of EC in the growing control group, a low outlier in the NT group, and the small sample size. However, *ANKH* shRNA might show significance with a larger sample size, and in particular, shRNA#1002 showed a trend for significance. However, the difference between the DOX-induced and control shRNA groups was highly significant (P = 0.005). The same vectors reduced *ANKH* mRNA expression from between 31% and 59% in IrrDSBsen in NHOF-1 cells (Supplementary Figures S3A, B, E, F), and EC was reduced by between 24% and 28% by all three shRNA vectors relative to the NT vector following induction by DOX (P = 0.03) and was reduced by 12% (vector #2500) and 27% relative to the non-induced controls (P = 0.025; Figure 1I). There was no significant effect of DOX on either *ANKH* mRNA or EC in the NT controls, EC in the medium blanks, or the cell counts in any of the experimental groups. There was also no significant reduction in p16^INK4A^ (Supplementary Figures S3C, G) and p21^WAF^ (Supplementary Figures S3D, H) mRNA levels or SA-βGal relative to the NT controls of shRNA vectors. However, vectors #1002 and #5474 showed a small but significant induction of p16^INK4A^ in growing cells only. Although the effect of shRNA knockdown on EC reduction was small, it was significant in IrrDSBsen cells, and given the modest level of *ANKH* mRNA knockdown, the data indicate that *ANKH* at least partially mediates EC accumulation following fibroblast senescence.

### 3.3 *ANKH* mRNA is upregulated along with senescence markers in PEsen human fibroblasts and is downregulated by the canonical function of telomerase

**FIGURE 2:**
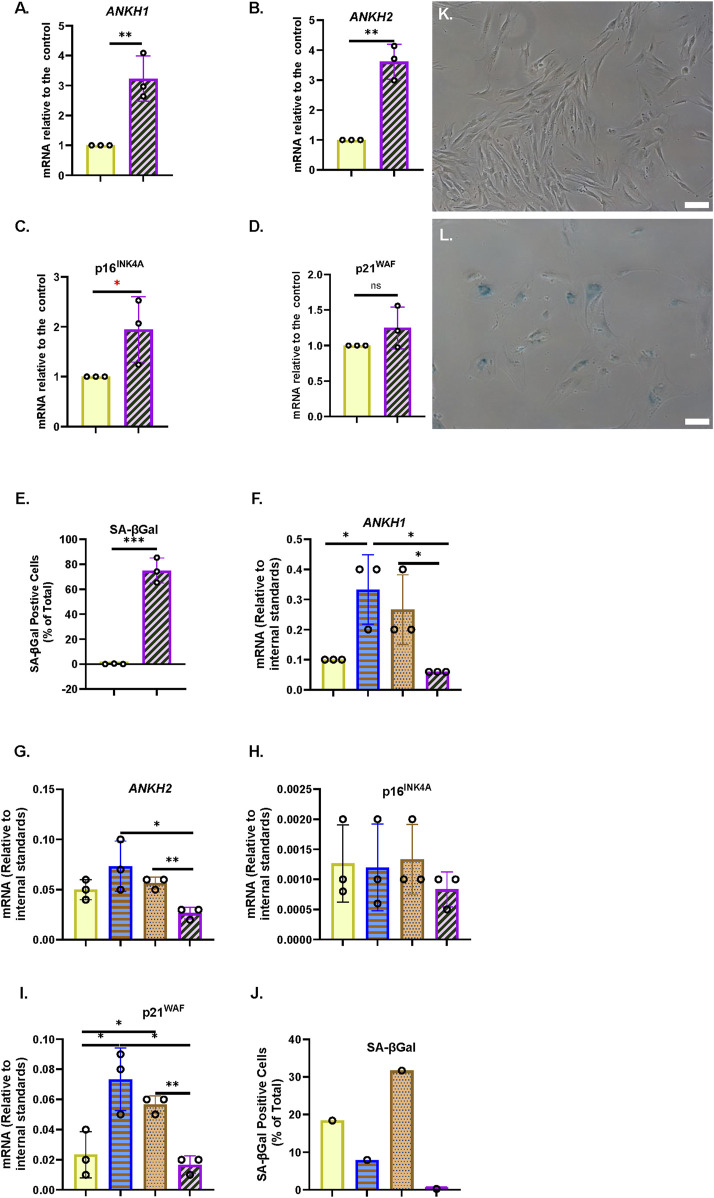
*ANKH* transcript is upregulated in PEsen human fibroblasts and downregulated by telomerase. **(A)** Expression of ANKH mRNA in growing and PEsen NHOF-1 cells using primer set 1. **(B)** This is similar to **(A)** but shows the results of primer set 2 in the same experiments. **(C)** This is similar to **(A)** but shows the expression of the senescence marker p16INK4A in the same experiments. **(D)** This is similar to **(A)** but shows the expression of the senescence marker p21WAF1 in the same experiments. **(E)** This is similar to **(A)** but shows SA-βGal expression. **(A–E)** Yellow plain bars, growing young cells 20.2-21 MPDs; blue horizontal striped bars, PEsen cells 68.2 MPDs. *, P < 0.05; **, P < 0.01; ***, P < 0.001; *P > 0.05 < 0.1; ns, not significant. The results are presented as the averages +/- standard deviation. N = 3. **(F)** Expression of ANKH mRNA in NHOF-1 PURO, TERT-HA, DNTERT, and TERT cell using primer set 1. N = 3. **(G)** This is similar to **(F)** but shows the results of primer set 2 in the same experiments. **(H)** This is similar to **(F)** but shows the expression of the senescence marker p16INK4A in the same experiments. **(I)** This is similar to **(F)** but shows the expression of the senescence marker p21WAF1 in the same experiments. **(J)** This is similar to **(F)** but shows SA-βGal expression. **(K,L)** Representative images of SA-βGal staining in **(E). (K)** Young growing NHOF-1 fibroblasts (20.2 MPDs). **(L)**. NHOF-1 cells (68.2 MPDs). Bar, 100 µm. **(F–J) (A–E)** Plain yellow bars, LATE PURO; blue horizontal striped bars, TERT-HA; brown stippled bars, DNTERT; and purple left-right diagonally striped bars, TERT all after completing 60 MPDs. *, P < 0.05; **, P < 0.01; ***, P < 0.001; * >0.05 < 0.1; ns, not significant. The results are presented as the averages +/- standard deviation. N = 4, except **(J)**.

Next, we tested whether *ANKH* was upregulated in PEsen human fibroblasts along with senescence markers and showed that this is, indeed, the case in NHOF-1 cells (Figures 2A,B); p16^INK4A^ (Figure 2C), p21^WAF1^ (Figure 2D), and SA-βGal (Figure 2E) are strongly upregulated in the same cells. Comparable results were obtained with PEsen BJ cells (Supplementary Figures S4A–E), except that p16^INK4A^ was not upregulated (Supplementary Figure S4C).

We have already reported that the canonical function of telomerase reduces the accumulation of EC along with bypassing senescence, so we next considered whether the transduction of NHOF-1 and BJ cells with *TERT*, but not the non-canonical variant *TERT-HA* (Stewart et al., 2002) or the dominant-negative (catalytically dead in fibroblasts) mutant *DNTERT* (Hahn et al., 1999), regulated the expression of *ANKH*. The results showed that in three independent NHOF-1 cultures and one BJ culture (Figures 2F,G; Supplementary Figures S4F, S4G), *TERT* expression reduced *ANKH* expression relative to the PEsen PURO controls. This occurred in parallel with p16^INK4A^ (Figure 2H), p21^WAF1^ (Figure 2I), and SA-βGal (Figures 2J–L; Supplementary Figure S4H). The *TERT-HA* and *DNTERT* controls, which fail to elongate telomeres (Counter et al., 1998; Hahn et al., 1999), did not reduce *ANKH* transcript levels or any senescence marker in either NHOF-1 (Figure 2) or BJ cells (Supplementary Figure S4). All *TERT* constructs were expressed in late passage NHOF-1 (Supplementary Figure S5A) and BJ cells (Supplementary Figure S5B). Only *TERT* increased telomerase activity in NHOF-1 cells (Supplementary Figure S5C), and only *TERT* and *TERT-HA* increased telomerase activity in BJ cells (Supplementary Figure S5D), as previously reported (Stewart et al., 2002).

### 3.4 *ANKH* mRNA and EC are regulated by the p38 mitogen-activated kinase, mitogen-activated protein kinase-activated protein kinase2/3, and the associated axis but not by the ataxia telangiectasia mutated kinase

**FIGURE 3:**
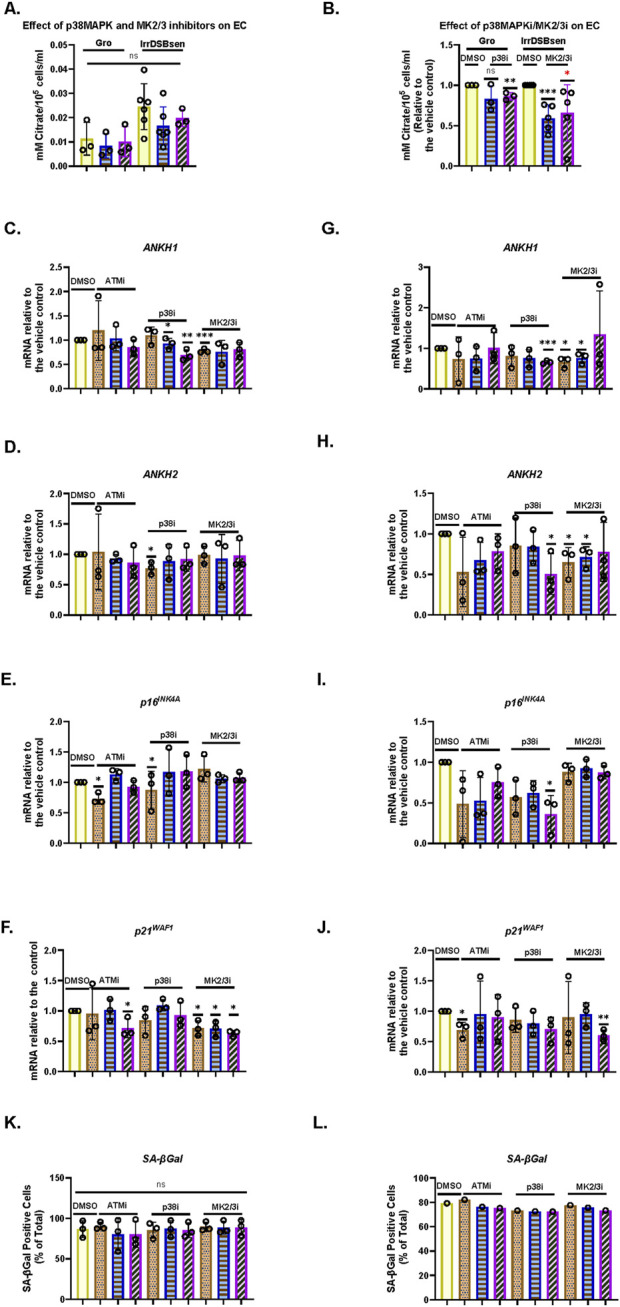
p38 mitogen-activated kinase, (*p38MAPK*)/MAPK-activated kinase2/3 (*MAPKAPK 2/3*), and *ATM* kinase regulate EC, secreted IL-6, and *ANKH* mRNA expression differently in human fibroblasts. **(A)** Effect of SB203580 (p38 inhibitor) and PF-3644022 (MK2/3 inhibitor) on EC in young growing and IrrDSBsen NHOF-1 cells (N = 3). Plain yellow bars, DMSO (vehicle) control; blue horizontal striped bars, SB203580 10 μM; purple left-right diagonally striped bars, PF-3644022 2.5 μM **(B)**. Same data as in A and a separate series of experiments but normalised to DMSO vehicle control (N = 6). **(C–L)** Effect of the kinase inhibitors SB203580 (p38MAPK), PF-3644022 (MK2/3), and KU55933 (ATM) on the ANKH transcript and senescence markers in IrrDSBsen cells. **(C–K)** BJ cells; **(G–L)** NHOF-1 cells. **(C)** and **(G)** Expression of ANKH mRNA using primer set 1. **(D)** and **(H)** Expression of ANKH mRNA using primer set 2. **(E)** and **(I)** Expression of p16INK4A mRNA. **(F)** and **(J)** Expression of p21WAF1 mRNA. **(K)** and **(L)** SA-βGal expression (%). Plain yellow bars, DMSO (vehicle) control; brown stippled bars, 1 μM; blue, horizontal striped; bars, 3 μM; purple left-right diagonally striped bars, 10 µm *, P < 0.05; **, P < 0.01; ***, P < 0.001; *P > 0.05 < 0.1; ns, not significant. The results are presented as the averages ± standard deviation (N = 3 except in **(B)**, where N = 6). The experiments in **(A,B)** and **(C.- L)** were performed at different times but using identical cell culture protocols and reagents.

We have previously reported that the kinetics of EC upregulation, following the induction of IrrDSBsen, are similar to that reported for the SASP and, in particular, interleukin-6 (IL-6) (James et al., 2018). As p38 mitogen-activated kinase (p38MAPK) (Freund et al., 2011), mitogen-activated protein kinase-activated protein kinase (MK2/3) (Laberge et al., 2015), and ataxia telangiectasia mutated (ATM (Rodier et al., 2009)) regulate the interleukins of the SASP, we tested whether the pharmacological inhibition of these kinases would regulate EC and *ANKH*. Figure 3 shows the effect of the p38MAPK and MK2/3 inhibitors, SB203580 and PF-3633022, respectively, on young, growing NHOF-1 cells and the same cells induced to senesce after the induction of IrrDSBsen. As reported previously, EC increased approximately 3-fold following IrrDSBsen and showed a trend for reduction with both drugs in both growing and IrrDSBsen groups, but the data were variable (Figure 3A). When the data were normalised to the controls for each experiment (Figure 3B), SB203580 showed a highly significant reduction in EC in IrrDSBsen cells but not growing cells, and PF-3633022 significantly inhibited EC in both growing and IrrDSBsen groups. Taken together, the data show that EC is regulated by both p38MAPK and MK2/3.

Next, we evaluated the effect of SB203580 and PF-3633022, as well as the *ATM* kinase inhibitor KU55933, on *ANKH* mRNA expression and senescence markers p16^INK4A^ and p21^WAF1^ over a larger dose range in both IrrDSBsen BJ (Figures 3C–K) and NHOF-1 cells (Figures 3G–L). The data showed that in both BJ and NHOF-1, SB203580 (between 1 and 10 µM) inhibited *ANKH* expression when assayed by both *ANKH* primer sets (*ANKH1* and *ANKH2*); PF-3633022 inhibited *ANKH* expression between 1 and 2.5 µM, although this was clearer with primer set *ANKH1*. The biphasic response of human fibroblasts to PF-3633022 regarding IL-6 secretion has been documented previously (Alimbetov et al., 2016), and the effect on *ANKH* mRNA expression was similar. KU55933 did not affect *ANKH* expression over this period. The abovementioned doses of inhibitors did not consistently affect the expression of p16^INK4A^ or p21^WAF1^, but 10 µM of SB-203580 reduced p16^INK4A^ expression in NHOF-1 cells (Figure 3I) and PF-3633022 reduced p21^WAF1^ expression in BJ cells at 1–10 µM (Figure 3F) and 10 µM in NHOF-1 (Figure 3J). None of the inhibitors affected the level of senescence, as assessed by the SA-βGal assay over the 3-day period of incubation in either BJ or NHOF-1 cells (Figures 3K,L).

Taken together, our data show that EC in IrrDSBsen cells is regulated along with IL-6 and *ANKH* by p38MAPK and MK2/3, independently of cell cycle arrest and the senescence phenotype but not by the *ATM* kinase.

### 3.5 EC is regulated by the p38 mitogen-activated kinase, mitogen-activated protein kinase-activated protein kinase2/3, and the associated axis, independently of cell volume, the ataxia telangiectasia mutated kinase, and IL-6

To evaluate the effect of SB203580 and KU55933 on EC and IL-6 more thoroughly, we exposed both growing and IrrDSBsen BJ cells to the drugs for 8 days individually and in combination. KU55933 alone had no effect on growing BJ cells, but SB203580, either alone or in combination with KU55933 dramatically increased the proliferation rate (Supplementary Figure S6A), reduced cell volume (Supplementary Figure S6B), and decreased SA-βGal (Supplementary Figure S6C), in line with previous reports showing that inhibiting p38MAPK antagonises cellular senescence (Iwasa et al., 2003). SB203580 and KU55933, but not KU55933 alone, also dramatically decreased EC (Supplementary Figure S6D), and the same combinations had similar effects in IrrDSBsen cells (Supplementary Figure S6D), which are likely due to SB203580 alone (see above). The same combinations did not affect IL-6 levels in growing BJ cells. However, KU55933 alone had a dramatic effect on IrrDSBsen cells, causing a many-fold induction in secreted IL-6, and this effect was almost ablated by SB203580, suggesting that the inhibition of ATM in senescent cells exacerbates signalling through p38MAPK to increase IL-6 under the conditions described in this study (Supplementary Figure S6E). Taken together, these datasets also highlight the fact that EC and IL-6 are independently regulated in senescent cells, which is further supported by the data below.

### 3.6 EC and *ANKH* are not suppressed by physiological levels of steroids and are regulated independently from interleukin-6 in IrrDSBsen human fibroblasts

**FIGURE 4:**
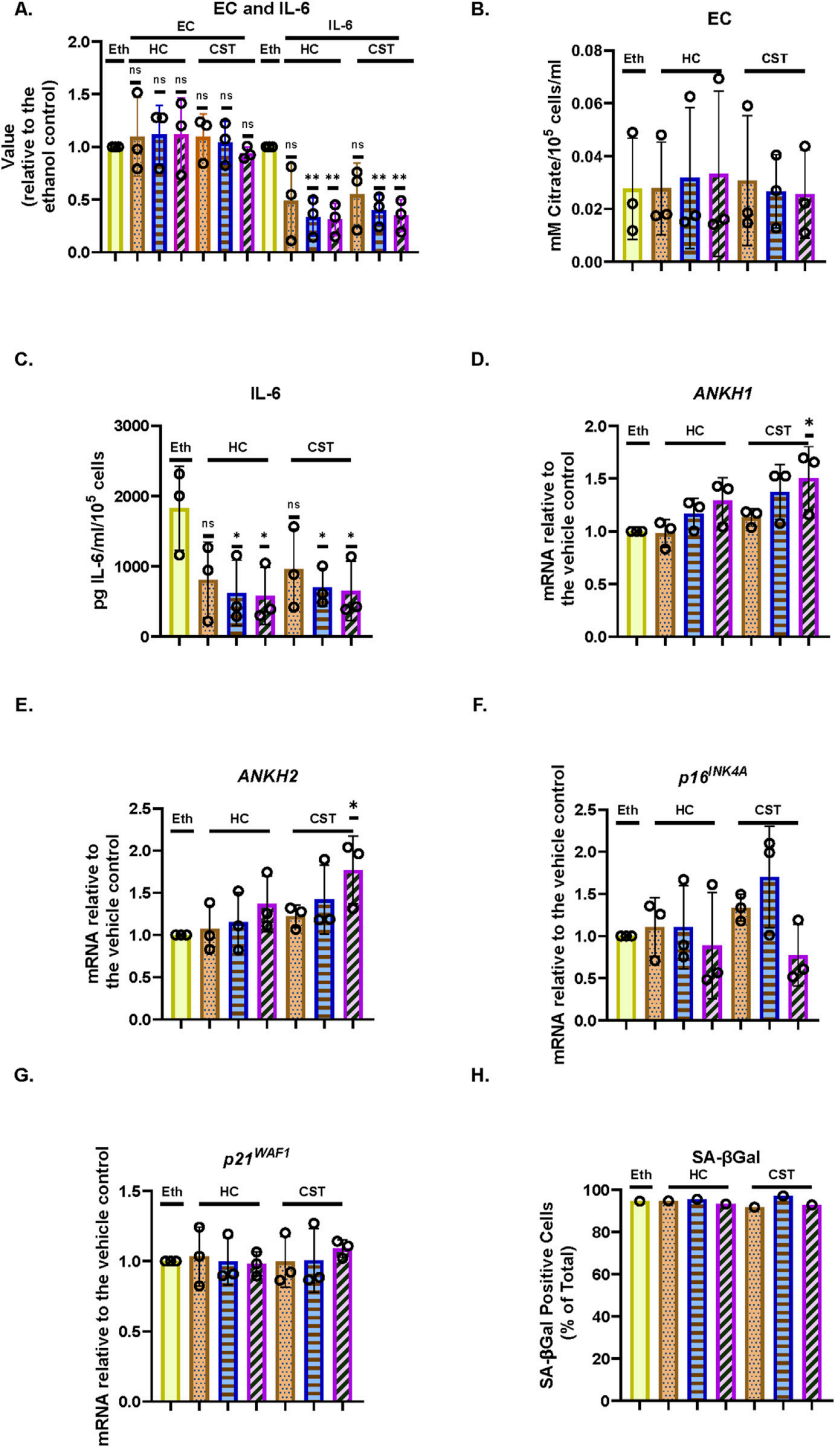
Effect of steroids on the EC, *ANKH* expression, and secreted IL-6 in IrrDSBsen human fibroblasts **(A)**. EC and IL-6 levels in the medium of IrrDSBsen BJ fibroblasts after treatment with the indicated doses of HC and CST (relative to the vehicle control). **(B)** Absolute EC levels in mM citrate per 10^5^ cells per mL from the experiment in **(A)**. **(C)** Absolute levels of IL-6 in pg/mL per 10^5^ cells from the experiment in **(A)**. **(D)** Effect of the indicated doses of HC and CST on the expression of ANKH mRNA using primer set 1. **(E)** This is similar to **(D)** but shows the results of primer set 2 in the same experiments. **(F)** This is similar to **(D)** but shows the expression of the senescence marker p16INK4A in the same experiments. **(G)** This is similar to **(D)** but shows the expression of the senescence marker p21WAF1 in the same experiments. **(H)** This is similar to **(D)** but shows the expression of senescence marker SA-βGal in the same experiments. Plain yellow bars, DMSO (vehicle) control; brown stippled bars, 1 μM; blue horizontal striped bars, 3 μM; purple left-right diagonally striped bars, 10 µm *, P < 0.05; **, P < 0.01; ***, P < 0.001; *P > 0.05 < 0.1; ns, not significant. The results are presented as the averages +/- standard deviation. **(A–G)** N = 3. **(H)** N = 1. The experiments in **(A–C)** and **(D–H)** were performed at separate times but using identical protocols and reagents.

It has been reported that both cortisol (hydrocortisone–HC) and corticosterone (CST) within the physiological range suppress many components of the SASP, including IL-6 (Laberge et al., 2012). This may explain why SASP factors are not that high in human plasma in clinical trials of senolytic drugs (Justice et al., 2019), during human chronological ageing (Stowe et al., 2010) or in the telomeropathy DC (James et al., 2023). We, therefore, tested the effect of HC and CST on EC, secreted IL-6 (Laberge et al., 2012), and *ANKH* expression over an 8-day period. In IrrDSBsen BJ cells, IL-6 is significantly reduced by HC and CST, as previously reported by others in a different setting (Laberge et al., 2012), but EC is not (Figures 4A–C). This supports the argument that EC and IL-6 are regulated independently. Interestingly, *ANKH* expression tended to increase, especially at high doses of CST in NHOF-1, but not BJ (Supplementary Figures S7A, B), rather than decrease (Figures 4D,E), whereas although EC increased slightly, this change did not reach significance. This anomaly may be due to the effects of CST on proteins other than EC and IL-6; there was no significant effect on p16^INK4A^ (Figure 4F), p21^WAF^ (Figure 4G), or SA-βGal (Figure 4H), and so, the steroid effects on IL-6 were independent of senescence. Comparable results were obtained with NHOF-1 cells (Supplementary Figure S7).

### 3.7 Interleukin-1 alpha inhibits the production of EC and *ANKH* expression in proliferating and IrrDSBsen human fibroblasts

**FIGURE 5:**
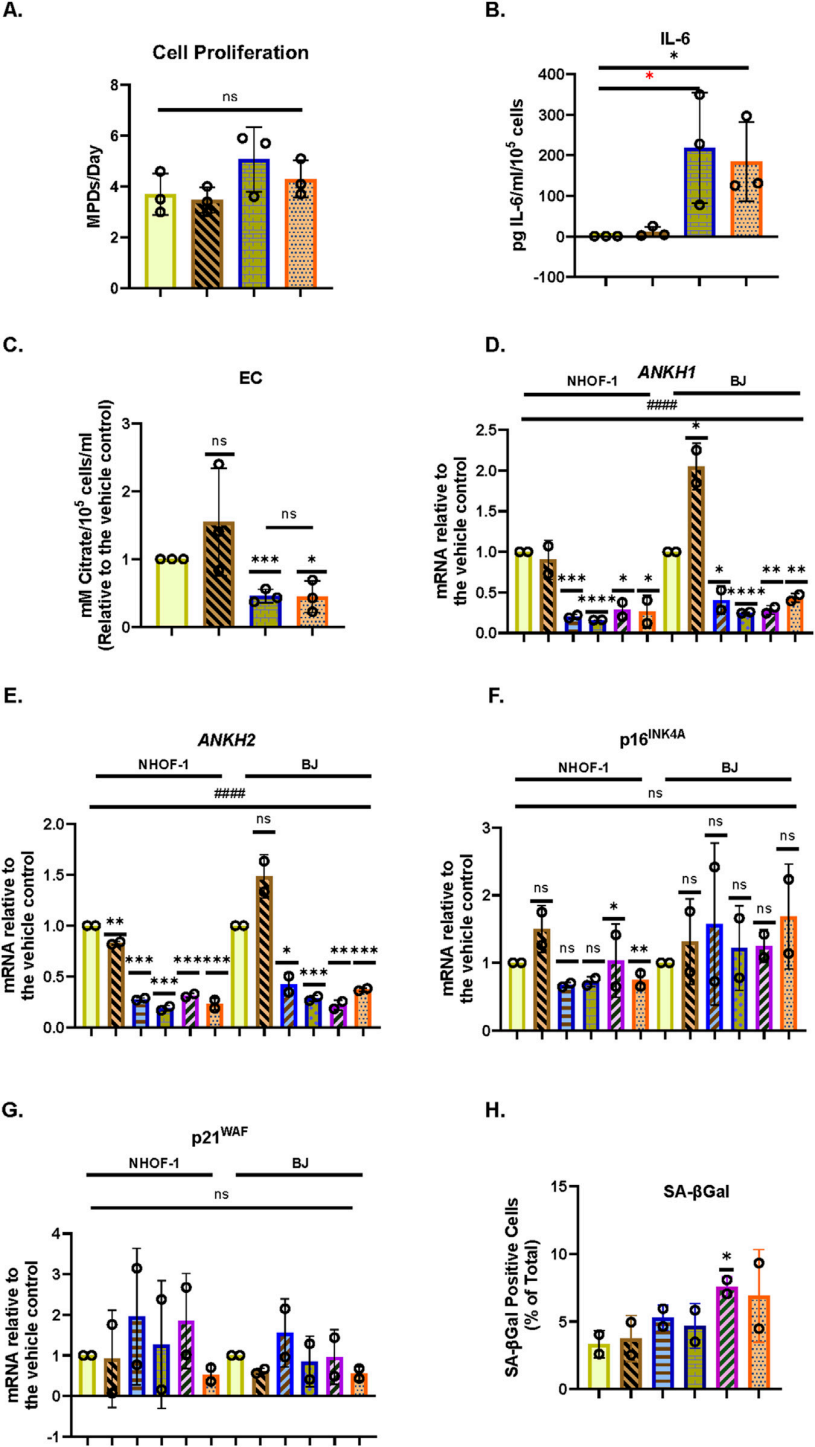
Effect of IL-1α on EC, *ANKH* expression, and secreted IL-6 in proliferating human fibroblasts. The figure shows the effects of an 8-day treatment with the indicated doses of IL-1α and HC on proliferating NHOF-1 and BJ cells. **(A)** Rate of proliferation of BJ cells in MPDs per day. **(B)** Absolute levels of IL-6 in pg/mL per 10^5^ cells in BJ cells. **(C)** Absolute EC levels in mM citrate per 10^5^ cells per mL in BJ cells. **(D)** The effect of the indicated doses of IL-1α and 300 nM HC on the expression of ANKH mRNA in BJ and NHOF-1 cells using primer set 1. **(E)** This is similar to**(C)** but shows the results of primer set 2 in the same experiments. **(F)** This is similar to **(C)** but shows the expression of the senescence marker p16INK4A in the same experiments. **(G)** This is similar to **(C)** but shows the expression of the senescence marker p21WAF1 in the same experiments. **(H)** Expression of senescence marker SA-βGal in the same BJ cell experiments. **(A–C)** Yellow bars, control; brickwork dark blue bars, 1 ng/mL IL-1α; right-left brown bars, 300 nm HC; stippled orange bars, 1 ng/mL IL-1α and 300 nm HC. **(D–H)** Plain yellow bars, control; horizontal light blue bars, 0.5 ng/mL IL-1α; brickwork blue bars, 1 ng/mL IL-1α; left-right diagonally striped purple bars, 2.5 ng/mL IL-1α; right-left diagonally striped brown bars, 300 nm HC; stippled orange bars, 2.5 ng/mL IL-1α and 300 nm HC. *, P < 0.05; **, P < 0.01; ***, P < 0.001; ns, not significant. The results are presented as the averages +/- standard deviation. **(A)** and **(B)** N = 3. **(C–G)** N = 2 for each cell line (N = 4 in total for both lines). All data were also analysed using ordinary two-way ANOVA (hatched lines). #, P < 0.05; ##, P < 0.01; ###, P < 0.001; ####, P < 0.0001; ns, not significant. The experiments in **(A–C)** and **(D–H)** were performed at different times but using identical cell culture protocols and reagents.

Next, we tested the effect of the SASP regulator interleukin 1 alpha (IL-1α), which was reported to positively regulate IL-6 (Laberge et al., 2012), on EC, secreted IL-6, and *ANKH* expression in BJ cells. Under the conditions described in this study, IL1α actually showed a slight trend for increased BJ proliferation in young cells (Figure 5A) along with a strong induction of secreted IL-6 (Figure 5B), as previously reported (Laberge et al., 2012), but reduced EC (Figure 5C) along with *ANKH* expression (Figures 5D,E) whilst having a minimal effect on p16^INK4A^ (Figure 5F), p21^WAF^ (Figure 5G), and SA-βGal (Figure 5H). We also tested the effect of IL-1α on IrrDSBsen fibroblasts (Supplementary Figure S8). IL-1α strongly stimulated the secretion of IL-6, an effect that was antagonised by HC, whereas the effect on EC was the reverse. In this separate set of experiments, HC alone also inhibited IL-6 secretion (Supplementary Figure S8A) but had no effect on EC (Supplementary Figure S8B).

### 3.8 The senescence inducer sodium butyrate induces IL-6 expression and proliferation arrest independently of EC

In addition, we tested another regulator of IL-6, sodium butyrate (NaB), which had been reported to induce secreted IL-6 independently of DNA double-strand breaks or senescence (Pazolli et al., 2012). Again, the data showed that in BJ cells and two oral fibroblast lines (NHOF-2 and NHOF-7), a four-day treatment of the cells with 1–4 mM NaB inhibited cell proliferation (Supplementary Figure S9A), caused cell flattening, increased cell volume, and induced IL-6 secretion (Supplementary Figure S9B), without significantly affecting histone acetylation (H3K27ac) (Supplementary Figure S9C) and collagen (Supplementary Figure S9D). 53BP1 levels were slightly increased in all cell lines but only significantly at 1 mM NaB (Supplementary Figure S9E), and SA-βGal levels were increased in BJ cells (Supplementary Figure S9F). In contrast, p16^INK4A^ (Supplementary Figure S9G) or p21^WAF1^ (Supplementary Figure S9H) mRNA expression was not increased. In all cell lines, EC was modestly and insignificantly induced over this time frame and not as high as in IrrDSBsen cells (Supplementary Figures S9I, J). In BJ cells, *ANKH* expression was, if anything, reduced (Supplementary Figures S9K, L).

These results confirm the association between EC and *ANKH* expression and the independent regulation of EC from IL-6.

### 3.9 EC and hydrogen peroxide are negatively regulated by p53 independently of *ANKH* expression and are independent of the senescence effectors p16^INK4A^ and p21^WAF1^

To evaluate the effect of p53 on EC regulation, we assessed a panel of human wild type, p53 null, and p21^WAF1^ null embryonic lung fibroblasts (Loxo26WT, Loxo26 p53−/−, and Loxo26 p21^WAF1^ −/−). The results showed that whether growing or following IrrDSBsen, Loxo26 p53−/− showed much higher levels of EC (Supplementary Figure S10A) and hydrogen peroxide (Supplementary Figure S10B) than their wild type and/or p21^WAF1^ null counterparts. qPCR data confirmed the absence of p53 in the p53−/− cells (Supplementary Figure S10C) and showed that p21^WAF1^ null Loxo26 cells had silenced p16^INK4A^, in addition to p21^WAF^, during passage after receipt (Supplementary Figures S10D, E). This means that EC regulation was independent of the senescence effectors p21^WAF1^ and p16^INK4A^. However, EC was restrained by p53, analogous to its reported regulatory function on the SASP (Supplementary Figure S10A; also refer to Rodier et al. (2009)).

The ability of p53 to restrain the accumulation of hydrogen peroxide (Supplementary Figure S10B) was consistent with its reported antioxidant function (Sablina et al., 2005), but under the conditions reported in this study, there was no effect of p21^WAF1^ and p16^INK4A^ on hydrogen peroxide levels. To evaluate whether the restraining effect of p53 on EC was mediated by *ANKH,* we assessed the cell line panel for *ANKH* expression but found no consistent upregulation of *ANKH* in p53−/− Loxo26 cells or p21^WAF1^/p16^INK4A^−/− Loxo26 cells (Supplementary Figures S10F, G). There was no dramatic effect of p53 deletion on SA-βGal levels (Supplementary Figure S10H) although p16^INK4A^ and p21^WAF1^ levels were significantly reduced (Supplementary Figures S10D, E). We also evaluated the effect of inducible *TP53* shRNAs on the expression of *ANKH* mRNA and EC in growing NHOF-1 fibroblasts. shRNA #2 reduced both *TP53* mRNA and its downstream target p21^WAF^ mRNA levels by 59% and 53%, respectively, relative to the NT control following induction by DOX (Supplementary Figure S10I) but did not lead to the upregulation of *ANKH* mRNA (Supplementary Figure S10I) or EC in these cells (Supplementary Figure S10J). *TP53* shRNA #2 significantly downregulated *ANKH* mRNA, which may indicate a role for *TP53* in regulating *ANKH* independently of EC. Taken together, the data suggest that *TP53* restrains EC independently of senescence or cell cycle inhibitors in certain fibroblast lines, analogous to its effects on the SASP, but may do so independently of *ANKH* expression.

### 3.10 Sodium butyrate induces senescence and EC accumulation independently of *ANKH* expression

To evaluate the effect of senescence inducers, other than those mediated by IrrDSBs or short telomeres, we assessed the ability of NaB to induce EC and senescence in the Loxo26 panel and p21^WAF1^/p16^INK4A^. We found that EC was elevated in the wild type cells following 3 weeks of 0.5 mM NaB treatment but not in BJ cells (Supplementary Figure S11A). This NaB effect was dependent on p53 and p21^WAF1^/p16^INK4A^. We then evaluated whether the effect on EC was associated with *ANKH* expression. We treated the Loxo26 panel, NHOF-1, BJ, and IMR90 cells for 17 days and observed the classical features of senescence, including cell flattening, increased cell size, increased p16^INK4A^ (Supplementary Figure S11B) and p21^WAF1^ (Supplementary Figure S11C) expression and increased SA-βGal in BJ and Loxo26 cells (Supplementary Figure S11D). However, despite inducing features of senescence, NaB did not induce *ANKH* expression in BJ, NHOF-1, or Loxo26 and only did so in IMR90 at high doses (Supplementary Figures S11E, F). Images illustrating increased cell size and flattening are shown in Supplementary Figure S11G–J, and those of SA-βGal are shown in Supplementary Figures S11I, J. This indicates that the induction of EC by NaB-induced senescence is variable between different fibroblast lines but is not in any case associated with *ANKH* induction.

### 3.11 Transforming growth factor β induces *ANKH* expression prior to inducing senescence

**FIGURE 6:**
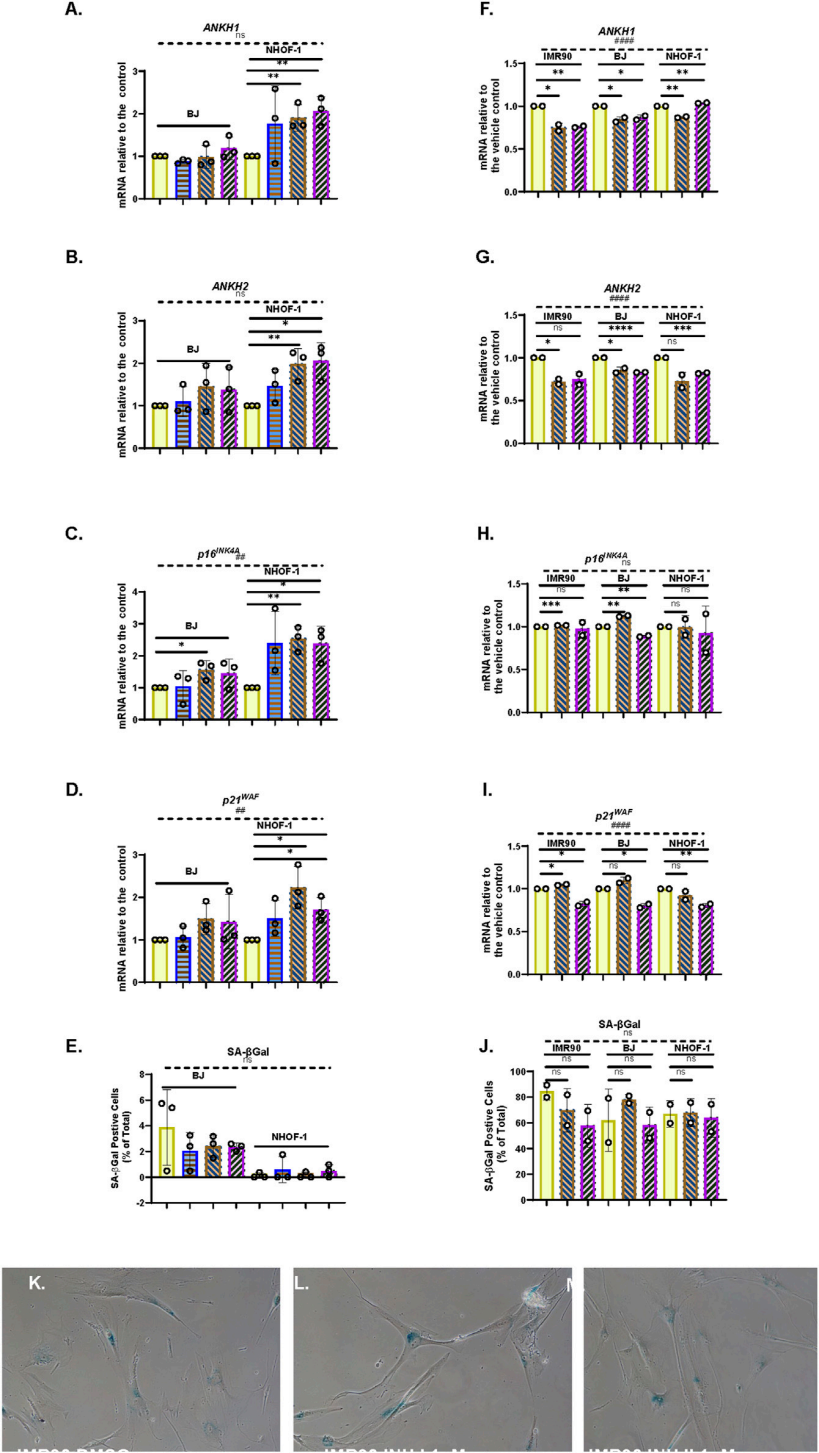
Effect of TGFβ on cellular senescence and the expression of *ANKH* in human fibroblasts **(A–E)**. Effect of TGFβ at doses of 1–10 ng/mL for 4 days on NHOF-1 and BJ fibroblasts on the expression of ANKH and senescence markers in growing young fibroblasts. **(A)** Expression of ANKH mRNA using primer set 1. **(B)** This is similar to **(A)** but shows the results of primer set 2 in the same experiments. **(C)** This is similar to **(A),** but shows the expression of the senescence marker p16INK4A in the same experiments. **(D)** This is similar to **(A)** but shows the expression of the senescence marker p21WAF1 in the same experiments. **(E)** This is similar to **(A)** but shows the expression of senescence marker SA-βGal in the same experiments. Plain yellow bars, control; blue horizontal striped bars, 1 ng/mL; brown right-left diagonally striped bars, 3 ng/mL; purple left-right diagonally striped bars, 10 ng/mL *, P < 0.05; **, P < 0.01; ***, P < 0.001; ns, not significant. The results are presented as the averages ± standard deviation. N = 3 for each line (N = 6 in total for all three lines). **(F–J)** Effect of TGFβ type I receptor kinase inhibitors on the expression of ANKH and senescence markers in IrrDSBsen IMR90, BJ, and NHOF-1 fibroblasts. **(F)** Expression of ANKH mRNA using primer set 1. **(G)** This is similar to **(A)** but shows the results of primer set 2 in the same experiments. **(H)** This is similar to **(A)** but shows the expression of the senescence marker p16INK4A in the same experiments. **(I)** This is similar to **(A)** but shows the expression of the senescence marker p21WAF1 in the same experiments. **(J)** This is similar to **(A)** but shows the expression of senescence marker SA-βGal in the same experiments. **(K–M)** Representative images of the SA-βGal staining of IMR90 cells in **(J)**. Plain yellow bars, vehicle control (0.1% DMSO); brown right-left diagonally striped bars, TGFβ type I receptor kinase inhibitor I at 1 µM; purple left-right diagonally striped bars, TGFβ type I receptor kinase inhibitor II at 1 µM. *, P < 0.05; **, P < 0.01; ***, P < 0.001; ns, not significant. The results are presented as the averages ± standard deviation. N = 2 for each cell line (N = 6 in total for all three lines). All data were also analysed using ordinary two-way ANOVA (hatched lines). #, P < 0.05; ##, P < 0.01; ###, P < 0.001; ####, P < 0.0001; ns, not significant.

TGF-β will induce reversible cell cycle arrest in many cell types (Shipley et al., 1986). The TGF-β family has also been implicated in the transfer of the senescence phenotype between cells (Acosta et al., 2013). To examine the effect of TGF-β on *ANKH* expression and senescence, we treated the cells for 4 days with 1–8 ng/mL of TGF-β1 in 4 mM HCl (Figure 6). The data showed that TGF-β1 significantly induced *ANKH* in NHOF-1 at low doses (Figures 6A,B) and showed a trend for induction in BJ cells (Figures 6A,B) along with increased p16^INK4A^ (Figure 6C) and p21^WAF1^ (Figure 6D) expression. However, there was no evidence of senescence, as assessed by SA-βGal (Figure 6E). These data indicate that TGF-β1 mediates *ANKH* expression along with fibroblast activation prior to or independently from senescence (Hassona et al., 2013). Next, to assess the role of the TGF-β family members in regulating *ANKH* and senescence markers in established IrrDSBsen fibroblasts, we treated three lines of IrrDSBsen fibroblasts with two TGF-β type I receptor kinase inhibitors that had been reported to suppress the SASP (Acosta et al., 2013). Figures 6F,G show that although the effect was small, both inhibitors significantly suppressed *ANKH* expression by 15%–30% in all three lines without reversing the levels of p16^INK4A^ (Figure 6H), p21^WAF^ (Figure 6I), and SA-βGal (Figure 6J), at least in the case of inhibitor I. However, there was a significant reduction in p21^WAF^ expression (Figure 6I) and a trend for SA-βGal reduction Figure 6J) in the case of inhibitor II. We conclude that the TGF-β family contributes to the regulation of *ANKH* in IrrDSBsen fibroblasts.

### 3.12 *ANKH* and EC are not upregulated in IrrDSBsen and PEsen keratinocytes or during keratinocyte differentiation, indicating cell type specificity in citrate export

As senescent mammary epithelial cells were reported not to shift their energy metabolism towards glycolysis and EC, albeit under very different culture conditions from those we used previously (Delfarah et al., 2019), we tested both EC accumulation and *ANKH* expression following IrrDSBsen in both 3T3-supported cultures and serum-free commercial media. We also tested the effect of the ROCK inhibitor Y27632 (ROCKi) in the 3T3 system, which has been reported to induce telomerase (Chapman et al., 2010), thus more accurately replicating conditions in the epidermal basal layer (Harle-Bachor and Boukamp, 1996). In all five epidermal keratinocyte lines, IrrDSBsen resulted in a downregulation of EC relative to controls (Supplementary Figure S12A), and the inclusion of ROCKi in the two 3T3-supported cultures did not change the result (Supplementary Figure S12B). We also evaluated the effect of PEsen on *ANKH* mRNA expression in regular 0.09 mM calcium medium after 4 and 7 days after the induction of differentiation and stratification by 0.4 mM and 1.0 mM of calcium chloride addition to the serum-free keratinocyte medium. There was no consistent increase in *ANKH* (Supplementary Figures S12C, D), whilst p16^INK4A^ (Supplementary Figure S12E) and p21^WAF^ (Supplementary Figure S12F) mRNA increased, as reported previously for p16^INK4A^ protein following PEsen (Loughran et al., 1996) in all conditions after 4 days, but not after the induction of differentiation (see below). We next addressed the role of differentiation and stratification in EC accumulation and *ANKH* expression in young cells by manipulating the calcium chloride concentration, as mentioned above, and monitoring the extent of differentiation through involucrin (*IVL*) and keratin 10 (*K10*) expression. Supplementary Figure S13 shows that in all five keratinocyte lines, there was no consistent increase in EC (Supplementary Figure S13A, B) following the induction of differentiation. In four of the five lines, there was no increase in *ANKH* (Supplementary Figures S13C, D), p16^INK4A^ (Supplementary Figure S13E), or p21^WAF^ (Supplementary Figure S13F) following the induction of differentiation, as assessed by IVL (Supplementary Figure S13G), or stratification by K10 (Supplementary Figure S13H). Taken together, our data show that there is no evidence that PEsen, IrrDSBsen, or terminal differentiation regulates either EC or *ANKH* expression in human epidermal keratinocytes.

### 3.13 *ANKH* mRNA is regulated in other IrrDSBsen and PEsen cell types relevant to ageing and age-related disease and chronologically aged mouse tissue

**FIGURE 7:**
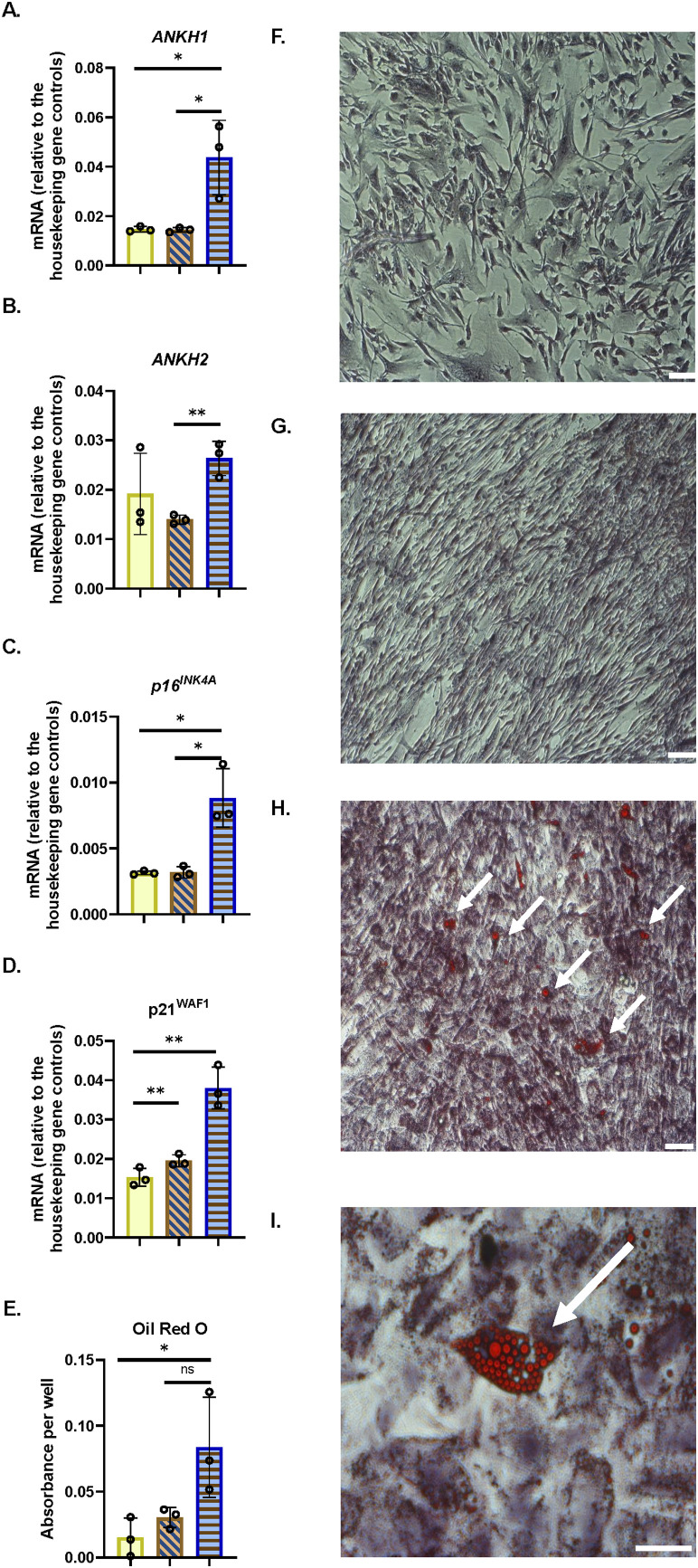
*ANKH* mRNA is upregulated in differentiated adipocytes following confluence-induced senescence. **(A)** Expression of ANKH mRNA using primer set 1. **(B)** Expression of ANKH mRNA using primer set 2. **(C)** Expression of p16INK4A mRNA. **(D)** Expression of p21WAF1 mRNA. **(E)** Oil Red O staining indicates adipocyte differentiation. Plain yellow bars, young growing control; brown right-left diagonally striped bars, young confluent (4 days control); blue horizontal striped bars, confluent (15 days) senescent differentiated adipocytes. *, P < 0.05; **, P < 0.01; ***, P < 0.001; *P > 0.05 < 0.1; ns, not significant. The results are presented as the averages +/- standard deviation. N = 3. **(F)** Young growing control. **(G)**. Young confluent control. **(H)** Young confluent differentiated showing cells with Oil Red-positive lipid droplets (arrows). Bar, 100 µM. **(I)** Higher power image of **(H).** Bar, 100 µM.

Next, we tested the relevance of *ANKH* upregulation in senescent cell types that are relevant to age-related disease in mice, such as astrocytes (cognitive decline (Ogrodnik et al., 2021)), adipocytes (frailty (Xu et al., 2018) and type II diabetes (Palmer et al., 2019)), and myoblasts (frailty (Zhang et al., 2022)). We now show that *ANKH* was upregulated in adipocytes following confluence-induced adipocyte differentiation (Figures 7A,B) and senescence (as assessed by high p16^INK11A^ and p21^WAF1^; Figures 7C,D) but not in PEsen or IrrDSBsen pre-adipocytes (Supplementary Figure S14). The levels of adipocyte differentiation in Figures 7A-7D were assessed by Oil Red staining (Figure 7E). Representative images of the cultures analysed in Figures 7A-7E are shown in Figures 7F-7I. We, and others, have shown that long-term quiescence induced by confluence can induce senescence (Munro et al., 2001; Marthandan et al., 2014).

Human myoblasts display markers of senescence and telomere-associated foci with increased chronological age (Zhang et al., 2022). Human myoblasts cannot be immortalised by telomerase alone, but we were able to compare senescent and pre-senescent human myoblasts (HMC) transduced with the catalytic subunit of telomerase, *TERT* (HMC *TERT* POOL or HMC *TERT* clone T2), and control (HMC) myoblasts. We were also able to compare these cells with a clone of *TERT*-transduced myoblasts that had spontaneously lost p16^INK4A^ (HMC *TERT* T15 (Wootton et al., 2003)). In senescent *TERT*-expressing cells, *ANKH* was upregulated following PEsen (Supplementary Figures S15A, B), along with p16^INK4A^ (Supplementary Figure S15C), p21^WAF^ (Supplementary Figure S15D), and SA-βGal (Supplementary Figure S15E). However, *ANKH* and all markers except p21^WAF^ were strongly downregulated following immortalisation in HMC *TERT* T15. These data also show that *ANKH* is associated with senescence in human myoblasts.

**FIGURE 8:**
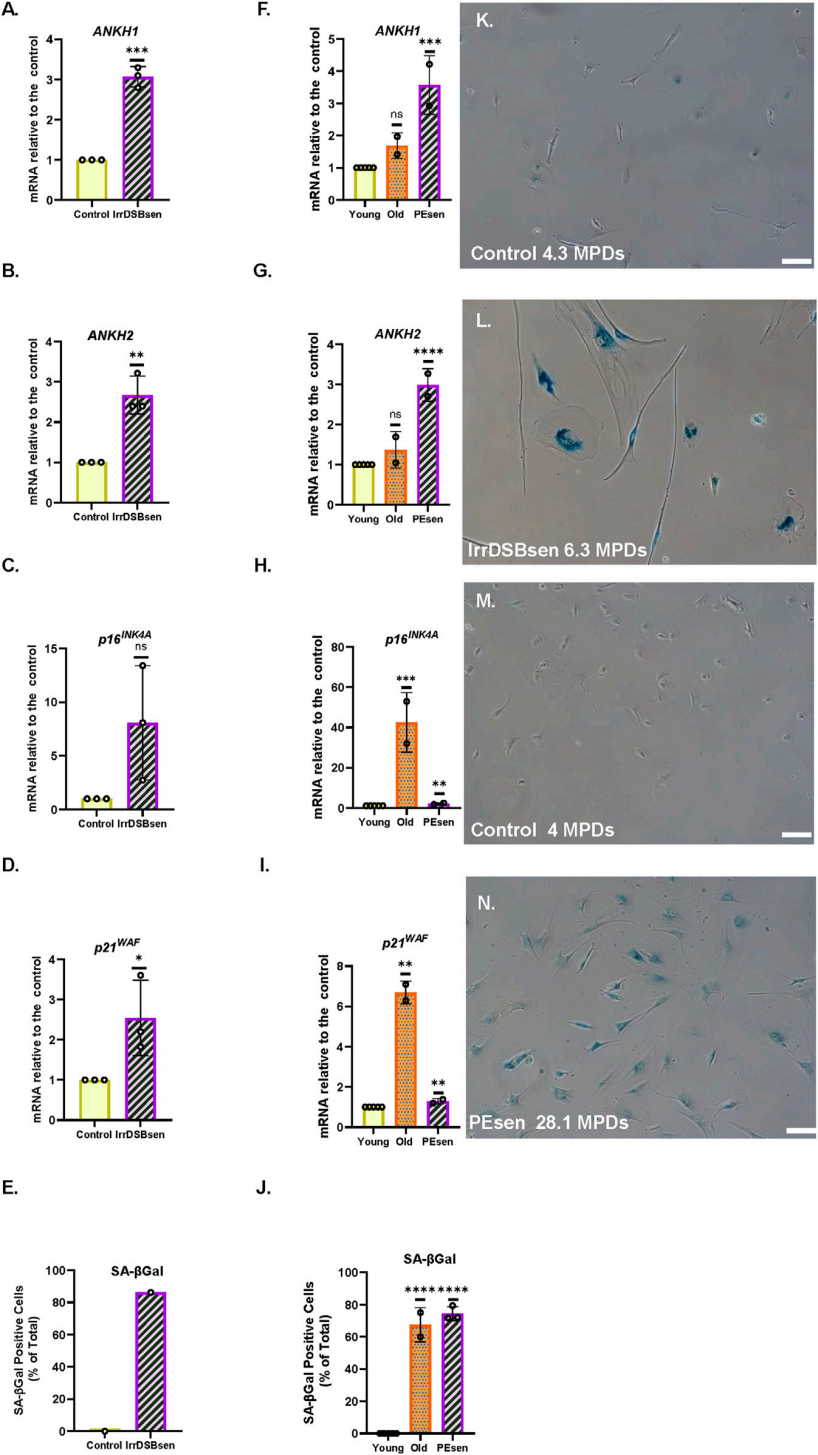
*ANKH* mRNA is upregulated in IrrDSBsen and PEsen astrocytes. **(A–E)** IrrDSBsen; **(F–J)** PEsen. **(A,F)** Expression of ANKH mRNA using primer set 1. **(B,G)** Expression of ANKH mRNA using primer set 2. **(C,H)** Expression of p16INK4A mRNA. **(D,I)** Expression of p21WAF1 mRNA. **(E,J)** SA-βGal expression (%). **(A–J)** Plain yellow bars, young growing control; purple left-right diagonally striped bars: **(A–E)** IrrDSBsen and **(F–J)** PEsen astrocytes (27–28.9 MPDs). **(F–J)** orange stippled bars, old (22–23 MPDs) astrocytes. *, P < 0.05; **, P < 0.01; ***, P < 0.001; ****, P < 0.0001; *P > 0.05 < 0.1; ns, not significant. The results are presented as the averages ± standard deviation. **(A–D)** N = 3; E is the result of a single experiment; **(F–J)** N = 2 (see also Supplementary Figure S16). **(K,L)** Representative images of SA-βGal staining in **(E)**; **(K)** young growing astrocytes (4.3 MPDs); **(L)** IrrDSBsen astrocytes (6.3 MPDs). **(M,N)** Representative images of SA-βGal staining in **(J)**. **(M)** Young growing astrocytes (4.0 MPDs); **(N)** PEsen astrocytes (28.1 MPDs). Bar, 100 µm.

Importantly, human astrocytes express *ANKH* (Figures 8A,B,G,H), and *ANKH* is overexpressed along with SA-βGal, p16^INK4A^, and p21^WAF1^ in IrrDSBsen (Figures 8A–E), as well as in aged and PEsen human astrocytes (Figures 8F–J, see also Supplementary Figure S16). Upon PEsen, the astrocytes became much flatter, lost their bipolar appearance, and increased in size (Figure 8N) compared to young astrocytes (Figure 8L). Several SASP cytokines (*IL1A*, *IL1B*, and *CXCL1*) were upregulated in PEsen astrocytes along with Complement Factor 3, indicative of astrocyte activation (Supplementary Figure S16). However, IL-6 was downregulated, and this may be due to its role in human astrocytes being replaced by IL-11, which has recently been reported to be increased in cellular senescence and relevant to certain ageing phenotypes in mice (Widjaja et al., 2024).

### 3.14 *Ank* is downregulated following mouse chronological ageing in parallel with senescence markers

**FIGURE 9:**
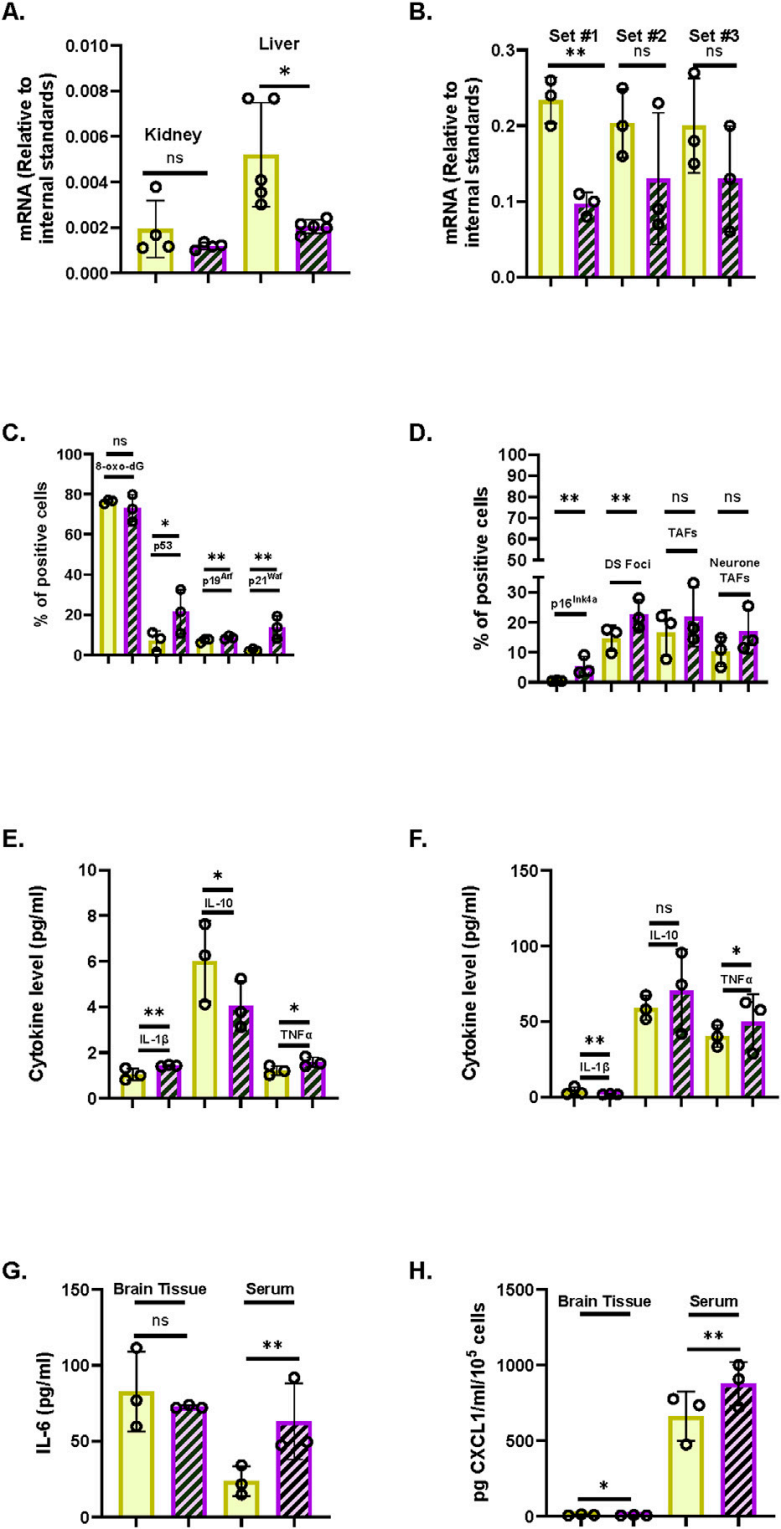
Aged mouse tissues show increased levels of senescence markers and inflammatory cytokines along with reduced levels of *ANKH* mRNA. **(A)** ANKH mRNA levels in young (4 months) and old (25 months) kidney (N = 4) and liver (N = 5) tissues. **(B)** ANKH mRNA levels in young (2.5 months) and old (17 months) brain tissues (N = 3). **(C)** Immunofluorescence staining of adult and aged C57BL/6 mice. **(A)** Percentage of cells expressing 8-oxo-dg, **(B)** percentage of cells expressing p53, **(C)** percentage of cells expressing p21Waf1, and **(D)** percentage of cells expressing p19Arf. **(D)** Immunofluorescence staining of adult and aged C57BL/6 mice. **(A)** Percentage of cells expressing p16Ink4a, **(B)** percentage of cells expressing γH2AX.53BP1 double foci (DF), **(C)** percentage of cells expressing TAF, and **(D)** percentage of neurones expressing TAF. **(E)** Different cytokine levels (pg/mL) in brain samples. Interleukin-1 beta (IL-1β), interleukin-10 (IL-10), and tumour necrosis factor alpha (TNF-α). **(F)** Different cytokine levels (pg/mL) in serum samples (interleukin-1 beta (IL-1β), interleukin-10 (IL-10), and (C) (E) tumour necrosis factor alpha (TNF-α)). **(G)** IL-6 levels (pg/mL) in brain and serum samples. **(H)** CXCL1 levels (pg/mL) in brain and serum samples. **(A,B)** Results analysed using the Student’s two-tailed unpaired test. *, P < 0.05; **, P < 0.01; ***, P < 0.001; ns, not significant. The results are presented as the averages +/- standard deviation. **(C–H)** Results represent the median, minimum, and maximum. Data were analysed using the Mann–Whitney U test; *p < 0.05. Three animals for adult C57BL/6, and three animals for aged C57BL/6. **(A)** Plain yellow bars, young (4-month-old mice); purple left-right diagonally striped bars, old (25-month-old mice). **(B–H)** Plain yellow bars, young (2.5-month-old mice); purple left-right diagonally striped bars, old (17-month-old mice).

**FIGURE 10:**
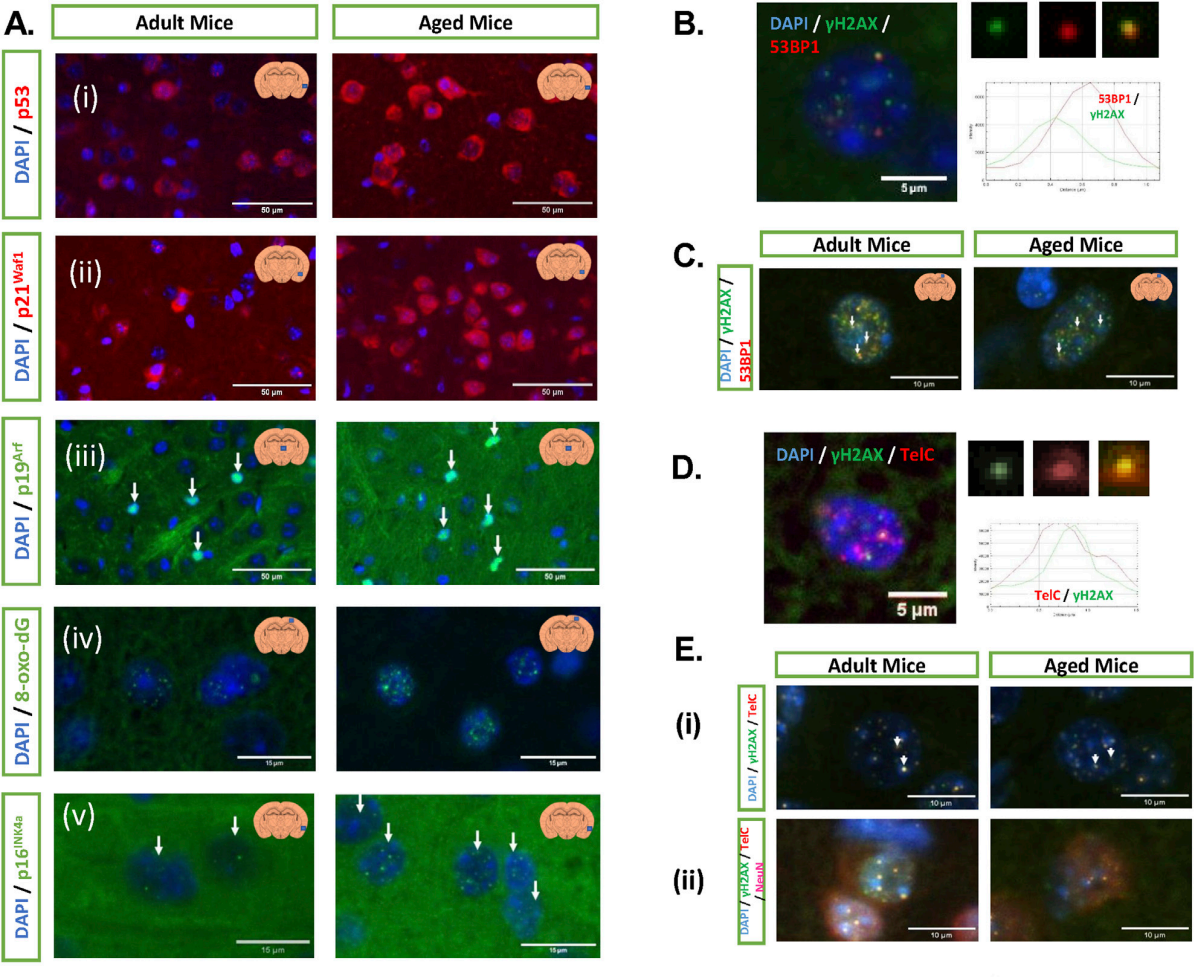
Immunofluorescence staining of multiple cellular senescence markers. **(A)** Representative images of the (i) p53 signal in the cerebral cortex, (ii) p21Waf1 signal in the cerebral cortex, (iii) p19Arf signal in the thalamus, (iv) 8-oxo-dG signal in the cerebral cortex, and (v) p16INK4a signal in the cerebral cortex. All images were taken at ×20 magnification. Bar, 50 µm. **(B)** Example of γH2AX and 53BP1 signals in a nucleus, the arrow demonstrates a DF, and the histogram displays their overlap. **(C)** Representative images of both groups. **(D)** Examples of TAF, γH2AX, and TelC signals in a nucleus, the arrow demonstrates a DF, and the histogram displays their overlap. **(E)** Representative images of TAF in a (i) nucleus and (ii) neurone. Images from **(B)** to **(E)** were taken at × 60 magnification. Bar, as indicated in the image.

To assess the role of *ANKH* (*Ank*) in mouse chronological ageing, we examined the levels of the *Ank* transcript in C57BL6 mouse tissues, where body fluid levels of citrate have been reported to be elevated at advanced chronological age. Figures 9A,B show that *Ank* is highly expressed in brain tissue relative to the liver or kidney. In all three types of tissue, there was a trend for decreased levels of the *Ank* transcript with chronological age, which was significant in the liver (Figure 9A) and the brain (Figure 9B). Older liver and kidney tissues showed increased *Ink4a* and *itgb3* transcript levels (Rapisarda et al., 2017), confirming the increased presence of senescent cells (Rapisarda et al., 2017). Regarding the brain, immunohistochemistry revealed increased levels of cells in older mouse tissues expressing p53, p19^ARF^, p21^WAF1^ (Figure 9C), p16^INK4A^, and ɤH2AX/53BP1) double-stained foci (Figure 9D). All aged brain cells (mainly microglia and neurones) and neurones alone showed a trend for increased telomere-associated foci with age (Figure 9D). Older mice showed increased levels of the SASP cytokines, such as IL-1β and TNFα; reduced levels of IL-10 in brain tissue (Figure 9E); and increased levels of IL-6, TNFα, and CXCL1 in serum (Figure 9F). Images of immunostaining in Figures 9C,D are shown in Figure 10. These results are consistent with the repression of EC and *ANKH* by IL-1α in human fibroblasts and the elevated levels of cytokines in the aged mouse serum and tissues (Rapisarda et al., 2017), including the brain (Figure 9E).

## 4 Discussion

There is now considerable evidence that energy (Fontana et al., 2018) and citrate uptake (Birkenfeld et al., 2011; Branco et al., 2022; Fan et al., 2021a; Fan et al., 2021b; Willmes et al., 2021; Fernandez-Fuente et al., 2023) are important for both ageing and age-related disease. For example, we previously reported that EC is regulated by the canonical function of telomerase *in vitro* and in a human disease characterised by telomere attrition, dyskeratosis congenita (DC), *in vivo* (James et al., 2023). This supports the hypothesis that senescent cell-derived EC may contribute to ageing and age-related disease, but the mechanisms underlying EC export are still unclear.

In this study, we demonstrate that EC and its plasma membrane transporter *ANKH*, but not other plasma membrane transporters, are associated with fibroblast senescence, independently of cell cycle arrest and cell size. However, the mitochondrial form of SLC25A1/mCiC is upregulated in some senescent cells, consistent with increased flux of citrate from the mitochondria to the cytoplasm. We have established that *ANKH* was required for EC accumulation in human fibroblasts using shRNA-mediated knockdown by three commercially validated inducible constructs. Furthermore, *ANKH* expression was tracked with EC across a wide range of *in vitro* experiments. This new information, together with previous work (Szeri et al., 2020), supports the hypothesis that *ANKH* is at least partially responsible for the observed EC accumulation in senescent human fibroblasts although other citrate transporters not yet identified may also contribute to EC accumulation in senescent cells.

However, the accumulation of EC in senescent cells and the increased expression of *ANKH* were cell-type-specific as neither occurred following keratinocyte or pre-adipocyte PEsen or IrrDSBsen or following keratinocyte differentiation and stratification. Nevertheless, increased expression of *ANKH* mRNA was observed in senescent astrocytes, differentiated adipocytes, and myoblasts as well as fibroblasts, and the increase in plasma EC in DC, where the telomeres of leukocytes and fibroblasts are short (James et al., 2023), implies that increased *ANKH* and EC are common to many cell types involved in ageing.

We previously established that the canonical function of telomerase downregulated EC along with senescence (James et al., 2023), and we have now established that *ANKH* is similarly regulated. As the kinetics of EC accumulation closely parallel that of the expression of IL-6 and other SASP proteins, we investigated the pathways reported to regulate IL-6 to test whether the same pathways also regulate EC. p38MAPK and its downstream kinase, MK2/3, were required for EC and *ANKH* transcript accumulation in established senescent cells, independently of cell size and cell cycle arrest under the culture conditions we used, although the effect was slightly less marked than that on IL-6. As p38MAPK is activated in numerous types of senescent cells, including astrocytes (Bhat et al., 2012), these results suggested that EC may also accumulate in these senescent cell types. TGF-β induced *ANKH* expression in some fibroblast types alongside senescence markers although the magnitude of this effect varied. However, as the TGF-β family was previously reported to spread the senescence phenotype in a paracrine fashion (Acosta et al., 2013), we tested whether pharmacologically inhibiting the TGF-β type 1 receptor kinase could downregulate *ANKH* in established IrrDSBsen fibroblasts. We found that this was the case although the effect was small and not associated with the reversal of senescence markers.

Taken together, our data suggest that EC accumulation via ANKH is limited to PEsen and IrrDSBsen and, perhaps, induced by TGF-β in certain cell types although the latter requires further investigation.

p53, which is known to restrain IL-6 in senescent pathways (Freund et al., 2011), also appeared to limit EC, but this effect varied with cell type and was independent of senescence and only partially associated with *ANKH* expression.

In addition, the histone deacetylase inhibitor NaB induced a large increase in cell size and an increase in IL-6, as reported by others (Pazolli et al., 2012), but did not induce significant levels of EC within 4 days. Furthermore, although longer-term NaB treatments induced senescence and, in some instances, EC accumulation, this was independent of *ANKH* induction, and so, *ANKH* does not mediate all forms of EC accumulation following senescence.

Importantly, we found that whilst the established orchestrator of the senescence-associated inflammasome, IL-1α (Acosta et al., 2013), induced high levels of IL-6 as reported (Laberge et al., 2012; Acosta et al., 2013) and its steroid inhibitors restrained it (Laberge et al., 2012), these manipulations had no effect on EC or *ANKH* in established senescent and proliferating cells. This last observation suggests that EC accumulation and *ANKH* are regulated independently from IL-6 and are not merely a consequence of the inflammasome or inflammation. IL-1α increased proliferation and reduced both EC and *ANKH* expression in the absence of any marked effect on senescence markers whilst at the same time increasing IL-6 secretion. These data further underline the differential regulation of EC and IL-6 secretion despite their similar kinetics and common regulation by the p38MAPK, MK2/3, and TGF-β pathways.

Astrocytes are the source of citrate supply in the nervous system although no candidate plasma membrane transporter has previously been identified (Mycielska et al., 2015). Our data, which show that *ANKH* is expressed by astrocytes, are significant as they suggest that *ANKH* is a candidate for a citrate exporter in astrocytes. Interestingly, we showed that *ANKH* is overexpressed in senescent human astrocytes *in vitro*. Moreover, the deletion of senescent astrocytes and microglia ameliorates tau-dependent pathology (Bussian et al., 2018) and senescent oligodendrocyte precursors’ amyloid beta pathology (Zhang et al., 2019) in mouse models of AD.

There was no evidence of an increase in the *Ank* transcript in three mouse tissues with chronological age, despite reported evidence of EC accumulation in several mouse biofluids with age. These data might suggest that *Ank* upregulation does not mediate EC accumulation in mice (Varshavi et al., 2018; Yue et al., 2022) but is consistent with *ANKH* and EC downregulation by IL-1α in human fibroblasts and with the upregulation of mouse SASP cytokines in the aged mouse brain we examined as in both situations, inflammatory cytokines such as IL-6 are elevated. Furthermore, single-cell RNA sequencing data of the aged mouse brain (Broad Institute Atlas of the Ageing Mouse Brain (Ximerakis et al., 2019)) showed considerable heterogeneity in *Ank* expression in different cell types. High levels of expression were observed in astrocyte and oligodendrocyte clusters, low levels in microglia, endothelial and vascular smooth muscle clusters, and variable expression in mature neurone clusters. These data are consistent with our new data obtained from aged microglial and neuronal tissues.

The role of *ANKH* in age-related disease in humans has yet to be determined, but genome-wide analysis has associated the *ANKH* gene with a risk of developing AD (Bellenguez et al., 2022; Tesi et al., 2024), other forms of dementia (Katsumata et al., 2022), and type II diabetes (Nugent et al., 2020). Furthermore, there is recent evidence linking *ANKH* downregulation in human vascular smooth muscle cells with aortic aneurism (Wu et al., 2024). The association of *ANKH* with AD is particularly interesting given that astrocytes are considered the major source of citrate supply to neurones in the brain (Mycielska et al., 2015). Moreover, we have shown that *ANKH* is upregulated in PEsen and IrrDSBsen astrocytes, which, in turn, are instrumental for AD development in mouse models (Bussian et al., 2018). However, we observed a downregulation of *Ank* in aged mouse brain tissue, mainly in neurones and microglial cells. Therefore, it is also possible that *ANKH* is linked to AD in humans (Bellenguez et al., 2022) is also related to its downregulation as the associated single-nucleotide polymorphism (rs112403360) is a non-coding intronic variant. Therefore, it is currently impossible to deduce how it pre-disposes to AD and the cell types in which it operates. However, cognitively healthy centenarians are enriched for the protective allele rs112403360. The protective role of the rs112403360 allele has been suggested to be related to its known role in bone and vascular health (Tesi et al., 2024), and there is evidence to support this (Wu et al., 2024; Pendleton et al., 2002; Katsumata et al., 2022; Nugent et al., 2020). However, the protective allele rs112403360 increased *ANKH* function in astrocytes; this could also benefit citrate supply to the brain. Significantly, loss-of-function mutations in the citrate plasma membrane importer, *SLC13A5*, in humans result in neonatal epilepsy, and the injection of citrate into the brain can induce seizures (reviewed in (2)), so citrate levels need to be tightly regulated as insufficient or excessive citrate uptake or production may be pathogenic.

EC is not merely a biomarker for cellular senescence and chronological age but induces inflammatory cytokines (Ashbrook et al., 2015) and participates in many age-related diseases in mouse models in a paracrine and systemic manner (see below). Conversely, IL-6, albeit at rather high doses can induce expression of the citrate importer *SLC13A5/INDY/Mi*ndy in both humans and mice (von Loeffelholz et al., 2017), and so, there is potential for a positive feedback loop between IL-6 and citrate uptake.

ANKH itself may not be a suitable drug target due to the reported deleterious effects of its dysfunction in both mice and humans (Szeri et al., 2020). However, the upstream negative regulators of EC and *ANKH* identified in this report, such as telomerase (Shim et al., 2024) and MK2/3 (Ozcan et al., 2015), are established drug targets that ameliorate senescence and age-related pathologies in mice. Furthermore, the recent identification of ATP citrate lysate as a target for excessive intracellular citrate accumulation following *Ank* loss-of-function (Wu et al., 2024) presents another pharmacological avenue to exploit citrate transport and *ANKH*/*Ank* depletion.

Inhibiting citrate importers such as *SLC13A5/INDY/Mindy* or *pmCiC* could be considered an approach to counteract the overexpression *ANKH*/*Ank*, where it occurs as drugs are being developed against both of these targets. However, pmCiC is expressed at a very low level in senescent fibroblasts. There are inhibitors of *SLC13A5/INDY/Mindy* that have beneficial effects in mouse models of age-related disease (Zahn et al., 2022). Furthermore, the *SLC13A5/Mindy* knockout mouse showed resistance to Type 2 diabetes (Birkenfeld et al., 2011), displayed lower blood pressure (Willmes et al., 2021) and a lower heart rate (Willmes et al., 2021) and, under certain circumstances, increased bone elasticity and strength (Zahn et al., 2023). *SLC13A5/INDY/Mindy* is not only expressed in the liver and neurones but also in the adrenal glands, and there is also evidence that high levels of plasma citrate due to *SLC13A5/INDY/Mindy* inhibition are nephroprotective (Gill et al., 2023). Targeted inhibition of *SLC13A5/Mindy* in the liver recapitulated many of the effects of the *SLC13A5/Mindy* knockout mouse on diet-induced nonalcoholic fatty liver disease (Brachs et al., 2016). A targeted mouse knockout study showed that the specific knockout of *SLC13A5/INDY/Mindy* in mouse neurones improved mouse memory, while a similar knockout targeting the mouse liver did not (Fan et al., 2021b). In contrast, citrate administration to mice fed on a high-fat diet improves memory by increasing ketogenesis (Fan et al., 2021a) and causes weight loss (Branco et al., 2022), but as predicted by earlier studies, it causes liver inflammation and insulin resistance and does not improve existing markers of type 2 diabetes (Branco et al., 2022). Citrate administration along with sucrose in mice fed with a conventional diet also induces markers of pre-diabetes (Leandro et al., 2016).

An additional issue is that the mouse *SLC13A5/INDY/Mindy* is different from its human paralogue having a different affinity and capacity for citrate (Kopel et al., 2021). Of even more concern is that loss-of-function mutations in human *SLC13A5/INDY/Mindy* do not lead to resistance to type 2 diabetes and instead cause neonatal epilepsy and several other developmental defects (Kopel et al., 2021). However, more recent human Mendelian randomization analysis using UK Biobank data indicated that SNPs linked to reduced *SLC13A5/INDY/Mindy* function lowered osteoporosis risk (Zahn et al., 2023) and also predicted improved kidney function (Gill et al., 2023). Therefore, *SLC13A5/INDY/Mindy* inhibition could benefit human health by compensating for *ANKH* upregulation in astrocytes but the reliance of human neurones on astrocyte-derived citrate (Mycielska et al., 2015) would advise caution until further research is conducted although designing SLC13A5 inhibitors that do not cross the blood–brain barrier would be possibility (Kopel et al., 2021).

## 5 Conclusion and future directions

In summary, our new data identifying that the novel citrate plasma membrane exporter *ANKH* is upregulated in a variety of senescent cell types relevant to ageing and its regulation by established drug targets inform on how countering the deleterious effects of senescent cells and telomere attrition might be addressed in the future. However, as *ANKH* and citrate may have opposing effects in humans and mice as well as in different ageing tissues, depending on dietary factors (Birkenfeld et al., 2011; Branco et al., 2022; Fan et al., 2021a; Fan et al., 2021b; Willmes et al., 2021), further research will be necessary to exploit these observations, especially in human tissues and organoids.

## 6 Limitations of the study

We have demonstrated a link between *ANKH* and EC upregulation in senescent human cells *in vitro* and offered a potential explanation for the downregulation of *Ank* in the aged mouse brain. However, although there is extensive evidence that *ANKH*/*Ank* regulates citrate export in both human cells and mouse tissues, the knockdown achieved by shRNA in human senescent cells was incomplete, and so, it remains to be established whether *ANKH* is the sole transporter mediating citrate export in human senescent cells. In future studies, the use of CRISP^R^ CAS knockout in cultured human fibroblasts and iPS cells subsequently induced them to differentiate into relevant lineages. As our *in vivo* data are currently only correlative, it also remains to be demonstrated that astrocyte Ank mediates the supply of EC to neurones and that its reduction mediates neurodegenerative pathologies such as AD. This could be addressed in future studies by targeted knockout of *Ank* in astrocytes as well as rescue experiments such as the overexpression or knock in of wild type *Ank*, as recently demonstrated in the role of *Ank* in aortic aneurism (Wu et al., 2024).

## Data Availability

The original contributions presented in the study are included in the article/Supplementary Material; further inquiries can be directed to the corresponding author.
